# Magnetized Cell‐Scaffold Constructs for Bone Tissue Engineering: Advances in Fabrication and Magnetic Stimulation

**DOI:** 10.1002/advs.202510094

**Published:** 2025-09-29

**Authors:** Elio Cinar SanSegundo, Mohammad J. Mirzaali, Lidy E. Fratila‐Apachitei, Amir A. Zadpoor

**Affiliations:** ^1^ Department of Biomechanical Engineering Faculty of Mechanical Engineering Delft University of Technology (TU Delft) Mekelweg 2 Delft 2628 CD The Netherlands

**Keywords:** bioprinting, bone tissue engineering, magnetic particles, magnetic stimulation, magnetized scaffolds, mechanotransduction

## Abstract

Magnetic particles (MPs), due to their unique physical and chemical properties, have emerged as promising tools in bone tissue engineering. Their incorporation into scaffolds or uptake by bone cells, combined with exposure to external magnetic fields, has been shown in various studies to enhance cell adhesion, proliferation, and osteogenic differentiation. In this review, the state‐of‐the‐art is presented on the synthesis processes of magnetized cells (MCs) and magnetized scaffolds (MSs), as well as the biological and mechanical effects of scaffold‐free MCs, cell‐seeded MSs, and MC‐seeded MSs under externally applied magnetic fields on bone tissue engineering. Furthermore, the specific applications of these systems is highlighted, such as non‐contact mechanical stimulation, and discuss their application to advance bone tissue engineering strategies.

## Graphical abstract

This review highlights the synthesis and applications of magnetized cells (MCs) and magnetized scaffolds (MSs) in advancing bone tissue engineering strategies. It further demonstrates how magnetic particles (MPs) improve scaffold properties and how external magnetic fields enhance mesenchymal stem cell adhesion, proliferation, and osteogenic differentiation, highlighting the significance of magnetomechanical stimulation.

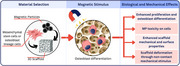

## 1 Introduction

Bone tissue engineering focuses on repairing critical‐sized bone defects, i.e., those too large to heal independently. Such defects, caused by trauma, non‐union fractures, infection, tumors, or bone atrophy, exceed the self‐repair capacity of bone tissue.^[^ ^1^ ^]^ Defects greater than 2 cm or involving over 50% of the bone circumference require intervention with filling materials.^[^ ^2^ ^]^ Currently, autografts and allografts are the most widely used strategies for treating these defects. However, these approaches present several limitations, including risks of disease transmission, donor site morbidity, and limited bone availability.^[^ ^3^ ^]^

To address these challenges, bone tissue engineering approaches are used that integrate four key components for achieving optimal bone cell growth: (stem) cells, scaffolds, biological cues (e.g., growth factors), and external stimuli.^[^ ^4^ ^]^ External (physical) stimuli, such as light, magnetic fields, ultrasound, electrical impulses, and direct mechanical forces, can prompt bone cell adhesion, proliferation, and differentiation within scaffolds by modulating their microenvironment.^[^ ^5^ ^]^ Advances in biomaterials have led to the development of polymers, ceramics, and composite scaffolds, which offer alternatives to traditional grafts by enhancing cell growth and osteogenic differentiation through their chemical and physical cues, in combination or not with external stimulation.^[^ ^1^ ^]^

Among external physical stimuli, magnetic fields have gained increasing attention for their potential to promote implant osseointegration and bone regeneration.^[^ ^6^ ^]^ Magnetic particles (MPs), particularly iron oxide nanoparticles, can be incorporated into cells or scaffolds to enhance specific properties.^[^ ^3^, ^7^ ^]^ Their integration into cells (magnetized cells, MCs) and scaffolds (magnetized scaffolds, MSs) has been widely studied, both individually and in combination, to assess their effects on cell adhesion, proliferation, and osteogenic differentiation under static (SMFs) or alternating magnetic fields (AMFs). When exposed to externally applied magnetic fields, MPs enable non‐contact mechanical compression of the scaffolds, mimicking the mechanical stress needed to stimulate cell differentiation and bone growth.^[^ ^8^ ^]^ This capability is particularly advantageous for implanted scaffolds without the application of an external force. In addition to mechanical stimulation of cells, MPs may improve the mechanical properties of the scaffolds, making them promising tools for bone regeneration.^[^ ^9^ ^]^ Furthermore, MPs have found applications beyond bone tissue engineering, including drug delivery, hyperthermia therapy, and biosensors.^[^ ^7^ ^]^

This review aims to provide a comprehensive overview of the role of MPs in bone tissue engineering. We first outlined the magnetic properties of iron oxide nanoparticles and existing synthesis techniques. Subsequently, we explored their integration into cells and scaffolds, and their individual and combined effects on bone growth, proliferation, and differentiation under magnetic fields. Finally, we discussed innovative strategies, such as MP incorporation into bioinks, and identified research gaps and limitations to guide future studies in the field.

## 2 Experimental Section

This literature review was conducted following the guidelines of the Preferred Reporting Items for Systematic Reviews and Meta‐Analyses (PRISMA) statement.^[^ ^10^ ^]^

### 2.1 Literature Search Strategy

Two primary research questions guided the literature review:

1. What are the intrinsic or induced magnetic properties of cells, and what techniques are used for their magnetization?
2. What are the effects of magnetization on scaffolds, cells, or their combination?

A systematic search was performed on April 01, 2025, using Medline (via PubMed) and Web of Science. Search queries, detailed in **Table** **1**, were developed using a combination of keywords and Boolean operators (e.g., “AND,” “OR”) to identify studies relevant to the research questions. The search was restricted to articles published in English.

**Table 1:** The search strategy used.

Search Topic	Database	Search Query	Results
Natural occurrence and induction of magnetic cells	Medline (via PubMed)	(”intrinsic magnetic*” OR ”magnetic cell*” OR ”cell magnetization”) AND (”magnetic nanoparticle*”) AND (”tissue engineering” OR ”bioprocess engineering” OR ”regenerative medicine”)	23
	Web of Science	AB = ((”intrinsic magnetic*” OR ”magnetic cell*” OR ”cell magnetization”) AND (”magnetic nanoparticle*”) AND (”tissue engineering” OR ”bioprocess engineering” OR ”regenerative medicine”))	16
Magnetic Nanoparticles in Scaffold‐Based Bone Tissue Engineering	Medline (via PubMed)	(”magnetic nanoparticle*”[Title/Abstract] OR ”Iron oxide nanoparticle*”[Title/Abstract] OR Magnetite[Title/Abstract] OR maghemite [Title/Abstract]) AND (scaffold [Title/Abstract]) AND (bone[Title/Abstract] OR osteoblast* [Title/Abstract] OR osteocyte* [Title/Abstract] OR osteoclast* [Title/Abstract])	89
	Web of Science	AB = ((”magnetic nanoparticle*” OR ”Iron oxide nanoparticle*” OR Magnetite OR maghemite) AND (scaffold) AND (bone OR osteoblast* OR osteocyte* OR osteoclast*))	114

### 2.2 Study Selection

Articles were screened based on predetermined eligibility criteria. Studies were included if they met the following conditions: *i*) involved the use of scaffolds for bone tissue engineering; *ii*) described magnetization of either the scaffold, the cells, or both; and *iii*) were original research articles written in English.

Exceptions were made for studies that did not utilize scaffolds or MPs but provided insights into magnetic (nano)particle interactions with cells or magnetic field interactions with cells. Studies were excluded if they met any of the following criteria: *i*) were not related to the topics of interest; *ii*) were exclusively in vivo studies; *iii*) were not written in English; or *iv*) did not have full‐text availability after reasonable efforts to obtain access.

### 2.3 Data Extraction and Analysis

The search results were exported to Zotero reference manager software (version 6.0.30), where duplicates were removed. Titles and abstracts were screened to identify potentially relevant studies, followed by a full‐text review to confirm eligibility based on the inclusion criteria.

From each eligible study, key data were extracted and compiled into a structured database. These data included scaffold preparation methods, materials or cell types used, MP synthesis methods, MP content, saturation magnetization, type and intensity of magnetic stimulation, and principal findings. This process ensured a comprehensive and systematic analysis of the literature, enabling a focused assessment of the effects of MPs on bone tissue engineering applications.

## 3 Results

Following the PRISMA guidelines, a total of 242 potentially relevant articles were identified from the database search. After eliminating 86 duplicates, 35 studies were excluded during the title and abstract screening phase. Subsequently, 121 studies were assessed in full, of which 14 were excluded for focusing on nanoparticles unrelated to the magnetic technologies of interest. To broaden the scope of the review, citation searching and exploration of additional sources (e.g., Litmap) added 33 more articles, resulting in a final selection of 140 studies.

In line with the search strategy, the selected studies were grouped into two major categories: 1) Studies investigating the natural occurrence or generation of magnetic cells to understand magnetism concepts. 2) Studies examining MPs within scaffolds for bone tissue engineering to assess the efficacy of scaffold‐based systems.

**Figure 1:**
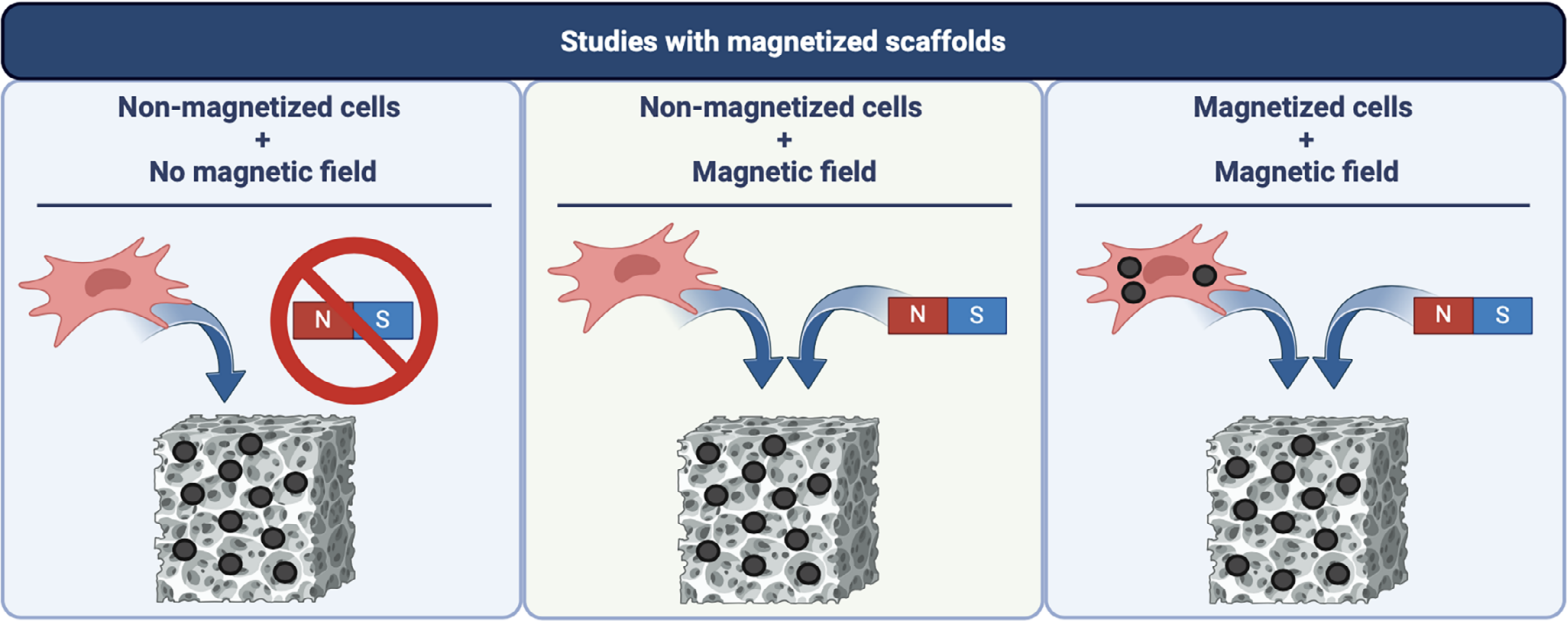
Main approaches and combination of studies encountered relating to magnetized scaffolds in bone tissue engineering (the gray porous structure represents the scaffold, and the black circles illustrate the MPs, not to scale).

Within the scaffold‐based studies, the experiments were further classified into three sub‐groups based on their experimental approaches: 1) MSs seeded with cells. 2) MS seeded with cells exposed to an externally applied magnetic field. 3) MS seeded with MCs under an externally applied magnetic field. This classification is summarized in **Figure** **1**.

### 3.1 Key Findings: Magnetic Cells and Scaffolds

*Magnetized cells* were primarily created by incorporating MPs into cells via endocytosis.^[^ ^11^ ^]^ The MPs were produced mostly by the co‐precipitation method, resulting in nanoparticles with uniform magnetization properties.^[^ ^12^ ^]^ These magnetized cells demonstrated enhanced osteogenic differentiation, with several studies attributing this effect to the activation of the mitogen‐activated protein kinases (MAPK) signaling pathway.^[^ ^13^ ^]^ The application of external magnetic fields further amplified these effects, suggesting a synergistic role between magnetic stimulation and intracellular MPs in promoting osteogenesis.^[^ ^14^ ^]^

*Magnetized scaffolds* were typically fabricated by incorporating MPs during scaffold production, which improved their mechanical properties and increased surface roughness.^[^ ^15^ ^]^ These enhancements were shown to facilitate better cell adhesion and proliferation within the scaffold.^[^ ^15^ ^]^ Notably, the addition of MPs to scaffolds also enabled non‐contact mechanical stimulation when exposed to an external magnetic field, mimicking the mechanical stresses required for optimal bone growth.

#### 3.1.1 MS Seeded with MCs Under Magnetic Fields

The approach based on MCs seeded on MS and exposed to external magnetic fields yielded the strongest outcomes, combining the intracellular effects of MPs in MCs with external mechanical stimulation induced by the magnetic field. These systems showed the highest levels of osteogenic differentiation, as evidenced by elevated expression of osteogenic markers and improved mineralization.

Across all studies, MPs incorporation enhanced both scaffold performance and cellular behavior, particularly under the influence of external magnetic fields. MP inclusion enhanced scaffold roughness and mechanical strength, key factors in promoting cell adhesion, spreading, and osteogenic differentiation. Additionally, external magnetic fields provided a non‐contact mechanical stimulation mechanism, further amplifying these effects. Collectively, these results highlight the potential of MPs in advancing bone tissue engineering strategies.

### 3.2 Magnetic Properties of Nanoparticles

#### 3.2.1 Magnetism

Incorporating MPs into scaffolds produces MSs, while integrating MPs into cells results in MCs. These magnetized components enable enhanced osteogenic differentiation and mechanical stimulation in bone tissue engineering under external magnetic fields. To understand their behavior, it is necessary to review the principles of magnetism at the nano‐ and micro‐scale. MPs are commonly composed of iron oxide nanoparticles, including magnetite (Fe_3_O_4_, Fe^2+^(Fe^3+^)_2_O_4_ and maghemite (γ‐Fe_2_O_3_, (Fe^3+^)_2_O_3_),^[^ ^16^, ^17^ ^]^ These materials are favored in tissue engineering due to their corrosion resistance, and safety under the conditions used for specific in vitro and in vivo applications.^[^ ^18^, ^19^ ^]^

**Figure 2:**
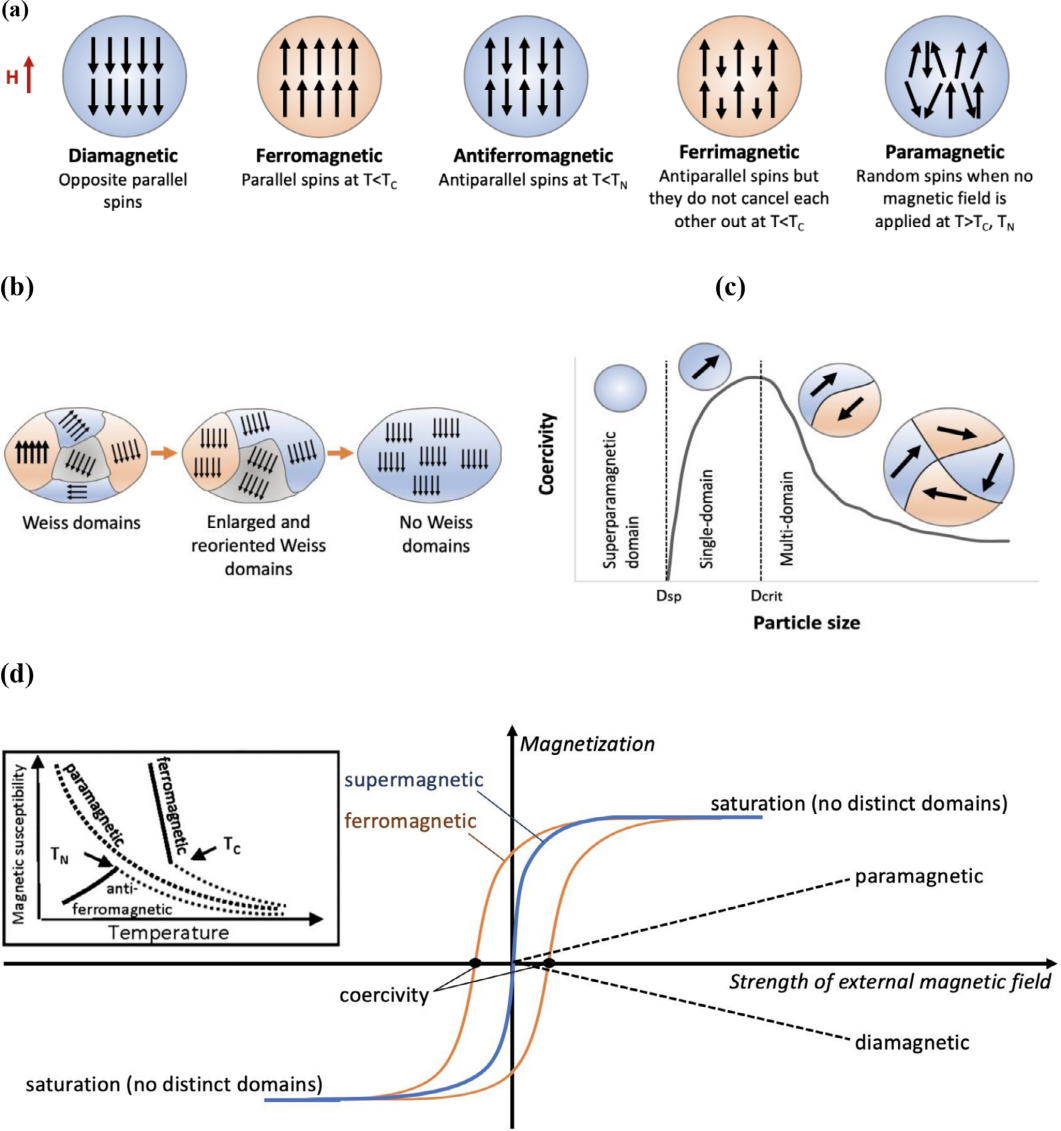
Illustration of diverse magnetic properties. a) Schematic alignment of magnetic moments (spins) for ferromagnetic, antiferromagnetic, ferrimagnetic, and paramagnetic materials according to the Curie‐temperature (*T_C_*) and Neel temperature (*T_N_*); b) widening and dissipation of Weiss domains in a ferromagnetic material as an externally applied magnetic field increases from left to right; c) coercive field versus nanoparticle diameter where *D_crit_* is the particle diameter where transition from multidomain to single‐domain and *D_crit_* is the diameter changing from single‐domain to superparamagnetic domain occurs; and d) Schematic representation of sample magnetization versus the strength of an external magnetic field. Since ferro‐ and ferrimagnetic materials typically exhibit similar curves, only ferromagnetism is depicted. Images modified with permission.^[^ ^20^ ^]^ Copyright 2020, Frontiers.

Materials’ magnetic behavior depends on the magnetic moments of electrons and their two spin states, and can be categorized into ferromagnetism, anti‐ferromagnetism, paramagnetism, and ferrimagnetism through unpaired electrons (unpaired spins) or diamagnetism through paired electrons (paired opposite spins)^[^ ^20^ ^]^(**Figure** **2**). These categorizations explain how their magnetization will behave under an exposed magnetic field. The formation of magnetic dipoles describes the nature of magnetic forces experienced by the exposed material.^[^ ^14^ ^]^

In the absence of a magnetic field, diamagnetic materials are known to have no net magnetization. Once a magnetic field is applied, they are magnetized in a direction opposite to the field, resulting in a repulsive force.^[^ ^21^ ^]^ In paramagnetism, the randomly oriented dipoles align to a certain degree, creating a low magnetization in the same direction as the magnetic applied field.^[^ ^21^ ^]^ In antiferromagnetism, magnetic dipoles align in an antiparallel manner, resulting in no net magnetization. Antiferromagnetic materials may transition to a paramagnetic state above a specific temperature known as the Curie temperature (*T_C_*) and Néel temperature (*T_N_*).^[^ ^21^ ^]^ In ferromagnetism, atoms arrange themselves in a lattice structure with atomic magnetic moments interacting and aligning parallel. Additionally, ferromagnetic materials exhibit magnetic domains, i.e., regions where atomic magnetic moments align uniformly in a single direction.^[^ ^21^ ^]^ Ferrimagnetism is only found in compounds exhibiting a combination of parallel and antiparallel alignments. Due to the difference in the alignment in both directions, a net magnetization can be found. However, this net magnetization is still lower than that found in ferromagnetism (Figure 2a).^[^ ^21^ ^]^ Iron shows ferromagnetism, whereas magnetite and maghemite exhibit ferrimagnetism.^[^ ^20^ ^]^

#### 3.2.2 Size Dependency in Magnetism

Magnetism is highly size‐dependent, particularly in nanoparticles, where properties differ significantly from bulk materials.^[^ ^22^ ^]^ The behavior transitions between multi‐domain, single‐domain, and superparamagnetic states as particle size decreases.

1. Multi‐domain state: In bulk materials, magnetic domains (Weiss domains) with differing spin orientations reduce the material's overall magnetization (Figure 2b).^[^ ^20^, ^23^ ^]^
2. Single‐domain state: Below a critical particle size (*D_crit_*) (e.g., 73 nm for magnetite^[^ ^24^ ^]^), particles exhibit uniform spin alignment, leading to higher magnetization. In the single‐domain state, two antiparallel orientations of the magnetic moment are favored. An energy barrier exists between these orientations, hindering a smooth transition of the magnetic moment from one stable equilibrium position to the other.^[^ ^3^ ^]^
3. Superparamagnetic state: At diameters below a secondary critical size (*D_sp_*) (e.g., 10 nm for magnetite^[^ ^24^ ^]^ and 15–18 nm for maghemite^[^ ^25^ ^]^), the energy barrier becomes comparable to thermal energy, facilitating the transition of magnetic moments between preferred orientations with an external field. Superparamagnetic nanoparticles show no remanent magnetization after the removal of the field, minimizing risks of particle aggregation (Figure 2c).^[^ ^3^ ^]^

Superparamagnetic MPs are particularly advantageous in biomedical applications, including bone tissue engineering, as they prevent long‐term residual magnetization while maintaining high magnetic responsiveness under an external field.

Temperatures also influence magnetic behavior. As the temperature rises above the *T_C_* or *T_N_*, materials transition from ferromagnetic or ferrimagnetic states to a paramagnetic state, losing their ordered spin alignment (Figure 2d). This property is critical when designing MPs for applications where temperature control is necessary, such as hyperthermia or tissue regeneration.

Magnetization curves provide additional insights into a material's magnetic properties. For example, diamagnetic and paramagnetic materials show linear curves with low magnetization, ferromagnetic and ferrimagnetic materials exhibit hysteresis loops, indicating coercivity and remanence due to Weiss domain movement. Superparamagnetic particles display reversible S‐shaped curves, with no hysteresis or coercivity, reflecting their unique combination of high magnetic susceptibility and rapid reversibility (Figure 2d).

#### 3.2.3 Synthesis Methods of Magnetic Nanoparticles

The size, shape, and surface characteristics of MPs are critical to their performance in tissue engineering.^[^ ^14^ ^]^ Two general approaches are used for MP synthesis: top‐down and bottom‐up methods.^[^ ^26^ ^]^ **Table** **2** summarizes their advantages and limitations.

**Figure 3:**
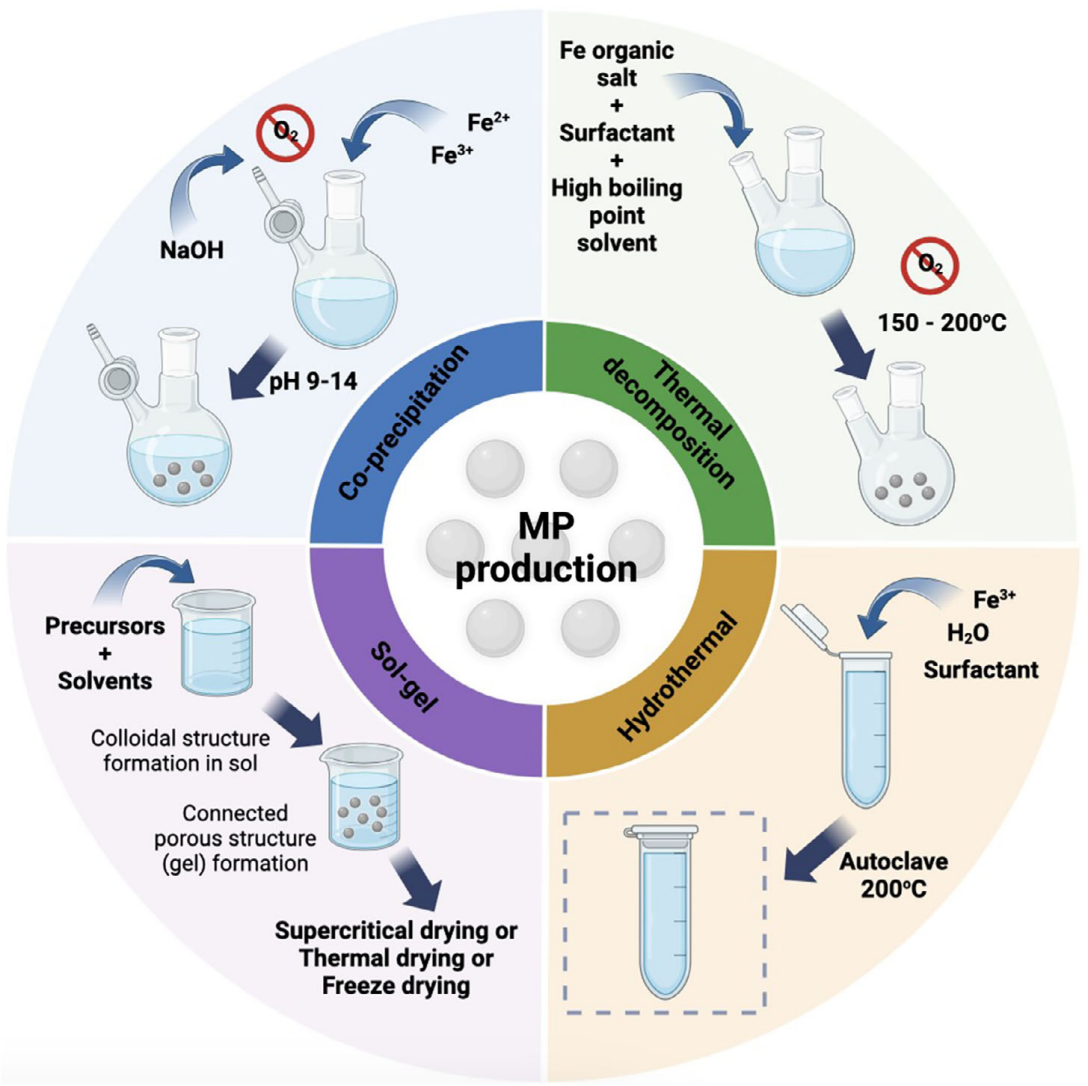
Overview of MP production methods, including co‐precipitation, thermal decomposition, hydrothermal, and sol‐gel methods.

1. Top‐down methods: These involve breaking down bulk materials into nanoparticles using techniques such as ball milling, laser ablation, or sputtering. While suitable for large‐scale production, top‐down methods often produce MPs with surface imperfections and limited control over size and shape.^[^ ^14^ ^]^
2. Bottom‐up methods (**Figure** **3**): These construct nanoparticles atom‐by‐atom, offering precise control over particle properties. Common bottom‐up techniques include:
3. Co‐precipitation: The co‐precipitation method involves nucleation and growth in a basic aqueous solution. For example, Sadeghzadeh et al., synthesized magnetite MPs (15–30 nm) by mixing ferrous and ferric salts under nitrogen at 80 °C, followed by ammonia addition and magnetic separation. The resulting MPs had a saturation magnetization of 65 emu g^−1^.^[^ ^12^ ^]^ While this method is simple and scalable, it is prone to agglomeration, which can be mitigated by using stabilizers,^[^ ^27^, ^28^ ^]^
4. Thermal decomposition: This technique uses organometallic precursors to produce highly crystalline and monodisperse MPs. H.‐M Yun et al. synthesized 10 nm magnetite MPs with a saturation magnetization of 4.8 emu g^−1^ (10% MP‐loaded scaffolds) by thermally decomposing Fe(acac)_3_ in phenyl ether with oleic acid and oleyl amine.^[^ ^29^ ^]^ This method allows precise control of particle size but requires high temperatures and specialized equipment.^[^ ^28^ ^]^
5. Hydrothermal method: This method uses high‐temperature, high‐pressure reactions. For instance, Sun et al. synthesized 260 nm Fe_3_O_4_ nanoparticles with a magnetic saturation intensity of 56 emu g^−1^ using a hydrothermal reaction at 200 °C for 12 h.^[^ ^30^ ^]^ Hydrothermal synthesis is straightforward but often produces larger, aggregated particles.
6. Sol‐gel method: The sol‐gel method involves hydrolysis or alcoholysis of metal alkoxides to form gels.^[^ ^31^ ^]^ Plan Sangnier et al. synthesized 8.8 nm magnetite MPs with a saturation magnetization of 51 emu g^−1^ by dissolving iron (III) acetylacetonate in benzyl alcohol and heating the mixture in a microwave reactor.^[^ ^32^ ^]^ This technique is versatile but requires careful control of drying and processing conditions to achieve desired properties.

**Table 2:** Advantages and disadvantages of the most commonly found bottom‐up MPs synthesis methods.^[^ ^28^ ^]^

Type of synthesis	Benefits	Drawbacks
Co‐precipitation	Convenient method, simple and rapid method, easy control of particle size and composition	Extensive agglomeration, poor morphology, and particle size distribution
Thermal decomposition	Producing highly monodispersed particles with a narrow size distribution	High cost, long‐time synthesis reaction, high temperature
Hydrothermal	High crystallinity, one‐step procedure, good dispersion in solution	Slow process, requires high temperatures, large particle size
Sol‐gel	Low processing cost, energy efficiency, high production rate, and rapid productivity	Limited efficiency, high cost, and contamination of MPs

Among these methods, co‐precipitation and thermal decomposition are the most prominent for tissue engineering applications due to their scalability and ability to produce MPs with desirable magnetic properties for specific applications.

Additionally, recent studies have explored the use of external magnetic fields during synthesis to influence nanoparticle crystal growth, morphology, and magnetic properties.^[^ ^33^ ^]^ While not the focus of this review, these field‐assisted synthesis approaches may offer complementary strategies for optimizing MP performance in tissue engineering applications.

### 3.3 Cell Magnetization in Bone Tissue Engineering

#### 3.3.1 Stromal Cells and Osteogenic Differentiation

Mesenchymal stromal cells (MSCs) are multipotent adult stem cells with the capacity to differentiate into various tissue lineages, including osteoblasts (bone), chondrocytes (cartilage), myocytes (muscle), and adipocytes (fat). MSCs can be isolated from multiple sources, such as bone marrow, adipose tissue, skeletal muscle, and synovial membranes.^[^ ^34^ ^]^ Among these, bone marrow‐derived MSCs are most frequently employed in bone tissue engineering due to their high proliferation rate, regenerative potential, and accessibility.^[^ ^35^, ^36^ ^]^ Apart from MSCs, embryonic stem cells^[^ ^37^ ^]^ and progenitor cells^[^ ^38^ ^]^ have also been utilized in studies involving MPs.

**Figure 4:**
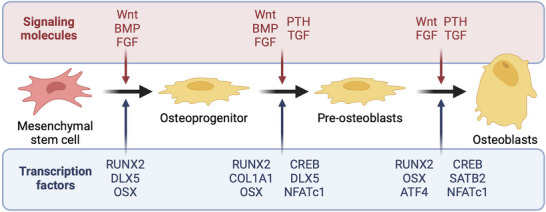
Stages of osteogenic differentiation from MSCs with the signaling molecules shown in pink and the transcription factors in blue. Image modified with permission.^[^ ^39^ ^]^ Copyright 2021, Biological Chemistry.

MSCs differentiate into osteoblasts under specific conditions, which are responsible for bone formation. Upon activation (i.e., often triggered by bone damage or compromised bone quality), osteoblasts synthesize key osteogenic components, such as collagen type 1 alpha 1 (COL1), alkaline phosphatase (ALP), and osteocalcin (OCN).^[^ ^39^ ^]^ These processes are regulated by key transcription factors, including runt‐related transcription factor 2 (RUNX2), distal‐less homeobox 5 (DLX5), and osterix (OSX), as well as signaling pathways like MAPK, Wnt, and BMP2/Smads (**Figure** **4**). Integrating MPs into cells offers a unique opportunity to activate these pathways through intrinsic and external magnetic fields, promoting osteogenesis and enhancing scaffold functionality.

#### 3.3.2 Uptake of Magnetic Nanoparticles by Cells

**Figure 5:**
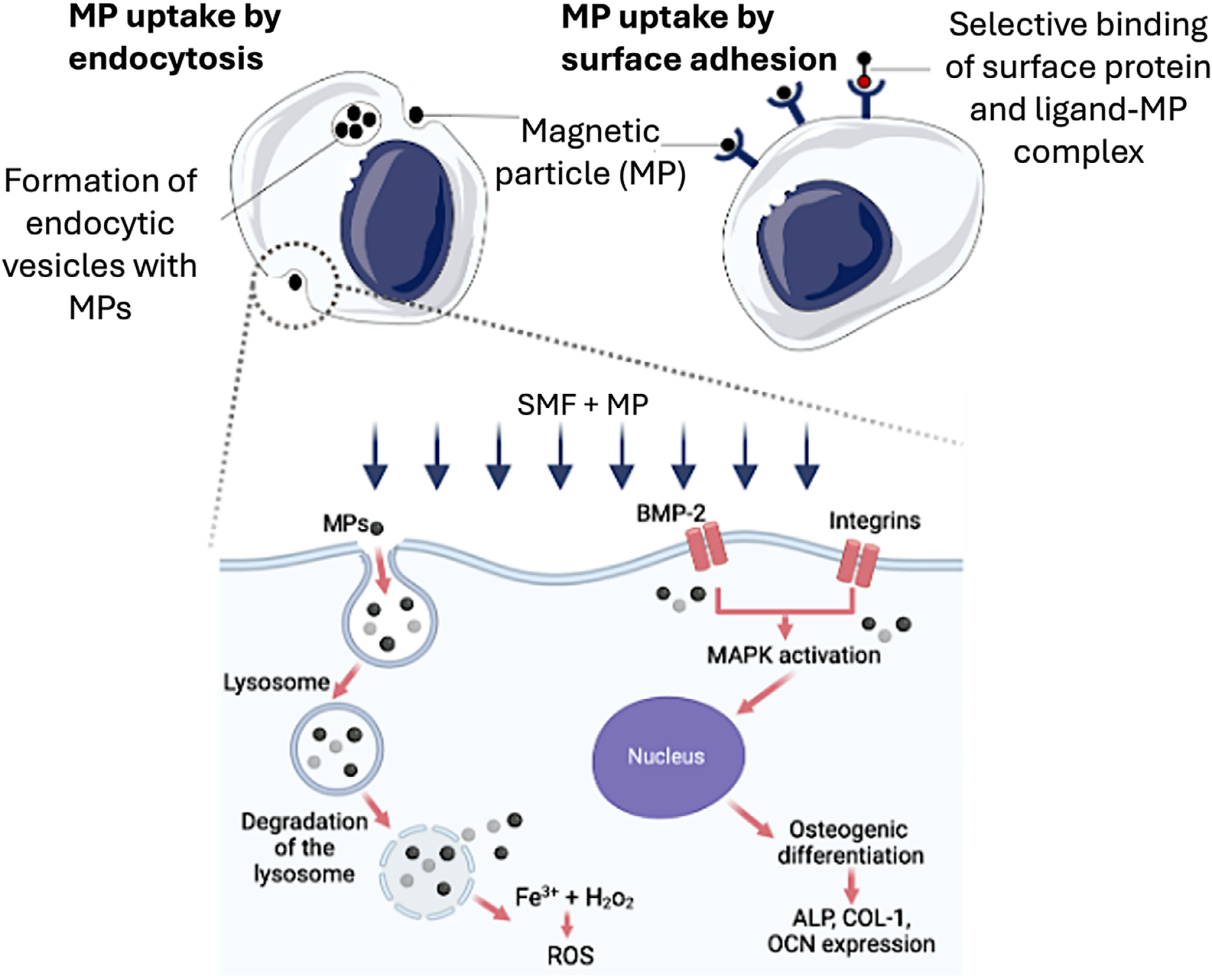
Effects of MP internalization by cells: (top) MP internalization by cells through (left) endocytosis; and (right) adsorption to the cell membrane or the attachment of a protein covalently linked to the MP to a cell surface.

MPs can interact with cells via endocytosis (internalization) or surface adhesion. For surface adhesion, MPs are often functionalized with bioactive molecules (e.g., proteins) to bind to specific membrane receptors (**Figure** **5**).^[^ ^20^ ^]^ However, endocytosis is the most widely reported mechanism of MP uptake.

The physiochemical properties of MPs, such as size, shape, surface charge, and coating, significantly influence cellular uptake.^[^ ^40^ ^]^ For instance, Xi Zhou et al. demonstrated that cube‐shaped MPs exhibited higher magnetization saturation (93 emu g^−1^) than octahedron‐shaped (89 emu g^−1^) or nanorod‐shaped particles (76 emu g^−1^), with magnetization decreasing as the elongation of nanoparticles increased.^[^ ^41^ ^]^ Coating strategies further enhance endocytosis; for example, Fayol et al. found that citrate‐coated MPs were fully internalized into MSCs and localized in endosomes, while uncoated MPs formed aggregates on the cell membrane.^[^ ^11^ ^]^

#### 3.3.3 Surface Coating Methods of Magnetic Nanoparticles

Due to their high surface energy and magnetic attraction, MPs tend to aggregate, which can compromise their safety and functionality in biomedical applications.^[^ ^42^ ^]^ Surface coating is therefore critical in achieving the required biocompatibility for the intended application.^[^ ^27^ ^]^ A wide range of coating strategies has been explored, including polymers (e.g., polyethylene glycol,^[^ ^43^ ^]^ chitosan^[^ ^44^ ^]^), organic surfactants, inorganic compounds (e.g., silica^[^ ^45^ ^]^), and bioactive molecules (e.g., growth factors).^[^ ^9^, ^45^, ^46^ ^]^

Surface coating either during or after particle synthesis plays a pivotal role in determining the intracellular fate and functional longevity of MPs within stem cells.^[^ ^14^, ^32^ ^]^ As demonstrated by Sangnier et al.,^[^ ^32^ ^]^ even when MNPs share an identical magnetic core, variations in surface chemistry, such as the type of chelating groups, polymer presence, and binding site density, profoundly influence their biological interactions. Notably, coatings rich in chelating functions, like polyacrylic acid (PAA), were associated with reduced degradation, while pre‐aggregation prior to cellular uptake further limited breakdown. This stabilizing effect of PAA coating was further validated in multilayered gelatin‐based systems fabricated by Samal et al.,^[^ ^47^ ^]^ where coated MNPs exhibited enhanced colloidal stability and uniform dispersion. The hydrophilic functional groups of gelatin interacted favorably with the coated particles, enabling consistent magnetic layering and predictable thermal behavior under magnetic fields. Additionally, Chen et al.^[^ ^48^ ^]^ demonstrated that superparamagnetic iron oxide nanoparticles (IONPs) coated with polyglucose sorbitol carboxymethylether (PSC) formed compact, uniform films on polymer scaffolds via layer‐by‐layer assembly. This coating not only improved hydrophilicity and mechanical interface properties but also enhanced protein adsorption and early‐stage cell adhesion. Importantly, the PSC coating facilitated “localized magnetic effects” that upregulated osteogenic markers such as ALP, RUNX2, and OCN, even in the absence of an external magnetic field. Together, these findings underscore that surface coating is not merely a passive interface but a strategic determinant of nanoparticle stability, cellular interaction, and bioactivity, which makes it a critical design parameter in regenerative nanomedicine.

#### 3.3.4 Magnetic Nanoparticle Toxicity

MP toxicity depends on factors such as composition, size, dosage, and oxidation state. For example, magnetite is more cytotoxic than maghemite due to its Fe(II)/Fe(III) ratio and oxidative potential.^[^ ^49^ ^]^ The optimal size range for biomedical MPs is 10–100 nm, balancing prolonged circulation times with effective clearance; particles smaller than 10 nm are eliminated via renal filtration, while those larger than 200 nm are captured by the spleen.^[^ ^49^ ^]^

Degradation of MPs releases iron ions, which can catalyze the production of reactive oxygen species (ROS). Excess ROS can lead to oxidative stress, DNA damage, and cell death.^[^ ^14^, ^45^, ^49^, ^50^ ^]^ Higher intracellular MP doses of 8 pg cell^−1^ and aggregated particles are associated with reduced degradation rates, increasing intracellular accumulation, and potentially exacerbating toxicity.^[^ ^32^ ^]^

#### 3.3.5 Influence of Cell Magnetization and Magnetic Fields on Bone Cells

##### Intrinsic Effects of Internalized MPs

MPs internalized by cells can generate intrinsic local magnetic microenvironments due to their spontaneous magnetic dipole alignments for brief moments, creating “transient micromagnetic fields”,^[^ ^51^ ^]^ which have been shown to significantly enhance osteogenesis.^[^ ^3^ ^]^ Without the need for external magnetic fields, the intrinsic momentary magnetization of MPs activates intracellular signaling pathways, most notably the MAPK pathway, which leads to increased expression of RUNX2 and BMP2.^[^ ^3^ ^]^ BMP2 subsequently activates Smads proteins, key signal transducers in the TGF*β* receptor family, which amplify RUNX2 expression and upregulate other osteogenic markers such as ALP, COL1, and OCN.^[^ ^52^, ^53^ ^]^ Moreover, internalized MPs have been observed to reduce intracellular hydrogen peroxide (H_2_O_2_), a common ROS, thereby promoting cell growth and reducing oxidative stress.^[^ ^44^ ^]^

The intrinsic magnetic field of superparamagnetic MPs, which do not exhibit residual magnetization, still facilitates osteogenesis by influencing intracellular ion channels, membrane potentials, and cytoskeletal dynamics.^[^ ^12^ ^]^ These effects enhance osteoblast differentiation while maintaining biocompatibility by preventing long‐term aggregation issues.

##### Combined Role of Internalized MPs and External Magnetic Fields

The application of an external magnetic field, particularly SMFs, further amplifies the osteogenic potential of magnetized cells. SMFs have been classified into weak (<1 mT), moderate (1 mT–1 T), and high (1–20 T) intensities,^[^ ^54^ ^]^ with moderate intensities (e.g., 400 mT) being the most widely studied. SMFs interact with internalized MPs to modulate mechanosensitive pathways, integrin signaling, and ion transport, leading to increased ALP activity, calcium deposition, and enhanced mineralization.^[^ ^3^, ^14^ ^]^ (Figure 5).

The combination of intrinsic magnetic fields (from MPs) and external SMFs has been shown to activate integrin‐mediated signaling pathways, such as the phosphorylation of focal adhesion kinase (FAK) and paxillin. These pathways are critical for cell adhesion, migration, and mechanotransduction, all of which are essential for scaffold‐cell integration.^[^ ^55^ ^]^ Furthermore, SMFs promote the deposition of extracellular matrix proteins, including COL1, and accelerate the fusion of bone cells with scaffolds, thereby improving bone regeneration. However, Huang et al., outlined that SMF‐induced differentiation is highly dependent on initial cell density, with lower densities responding more favorably to magnetic stimulation.^[^ ^56^ ^]^

##### Independent Effects of Magnetic Fields Without Magnetic Particles

Several studies have demonstrated that SMFs or AMFs alone can influence bone cell behavior, even in the absence of MPs. Early studies showed that osteoblastic MC3T3‐E1 cells exposed to a high 8 T SMF for 60 h became rod‐shaped and aligned parallel to the magnetic field direction, showing that SMFs can control cellular orientation.^[^ ^57^ ^]^ Although proliferation wasn't affected as much through the application of SMFs to cells, ALP activity and matrix mineralization increased, indicating enhanced differentiation and osteogenic behavior.^[^ ^57^, ^58^ ^]^

Y. Xia et al., investigated human dental pulp stem cells cultured in MP‐containing media (5 µg mL^−1^) and exposed to SMFs. Their study demonstrated that osteogenic differentiation markers (ALP, RUNX2, COL1, and OCN) were significantly upregulated when MPs and SMFs were combined, compared to controls without MPs or SMFs.^[^ ^59^ ^]^ Notably, the study found no adverse effects on cell proliferation, affirming that magnetic conditions support both growth and differentiation.

Zablotskii et al. provided a theoretical framework for SMF effects, demonstrating that a moderate‐strength magnetic field (≈1 T) coupled with a large magnetic gradient (up to 1 GT m^−1^) can alter the membrane potential of cells, influencing cellular behavior and fate.^[^ ^60^ ^]^ Such changes are believed to enhance the responsiveness of mechanosensitive receptors and promote osteogenesis.

AMFs, though less commonly studied in bone tissue engineering, may offer complementary advantages. AMFs can produce dynamic magnetic forces on internalized MPs, potentially enhancing intracellular signaling and promoting more uniform cell proliferation and differentiation.^[^ ^61^ ^]^ Future studies should compare SMFs and AMFs in the context of MP‐based scaffolds to determine their relative efficacy.

Clinically, these findings highlight the potential of combining magnetized scaffolds and externally applied SMFs to enhance implant integration and bone healing. For example, custom‐designed scaffolds seeded with MSCs pre‐labeled with MPs could be paired with wearable SMF devices to provide localized, non‐invasive mechanical stimulation in patients with critical‐sized bone defects.

### 3.4 Scaffold Magnetization in Bone Tissue Engineering

Scaffolds are fundamental in bone tissue engineering, serving as 3D frameworks that support cell attachment, proliferation, and differentiation in an environment that mimics the natural extracellular matrix. Integrating MPs into scaffolds to create MSs has gained significant attention due to their ability to stimulate osteogenesis through intrinsic magnetic fields, enhanced mechanical properties, and compatibility with external magnetic fields under specific conditions.

This section explores the biomaterials and production methods used to fabricate MSs, the effects of MP incorporation on scaffold properties, and how scaffold magnetization influences cellular behavior with and without external magnetic field exposure.

#### 3.4.1 Biomaterials Used in Magnetized Scaffolds

The choice of biomaterials is critical to scaffold functionality in bone tissue engineering. Scaffolds must meet specific criteria, including cytocompatibility, biodegradability, suitable mechanical properties, and optimal porosity to support cellular activity. These properties are essential to create an environment conducive to bone regeneration.^[^ ^34^, ^62^ ^]^ A summary of the benefits and drawbacks of these biomaterials is provided in **Table** **3**.

**Table 3:** Overview of the benefits and drawbacks of the biomaterials used for bone tissue engineering (table modified with permission.^[^ ^93^ ^]^ Copyright 2014, Springer Nature).

Biomaterials	Benefits	Drawbacks
Ceramics (e.g., Bioglass, HA, TCP, and related calcium phosphate)	Supporting cell activity; good osteoconductivity; vascularization; adaptable degradation rate	Brittle; slow biodegradation in the crystalline phase
Natural polymers	Low toxicity; bioactivity; biodegradability	Low mechanical, thermal, and chemical stability; possibility of immunogenic response
Synthetic polymers	Biodegradability; bioresorbable; good processability; good ductility	Inflammation caused by acid degradation products; Limited mechanical properties; Slow biodegradability

##### Ceramic Biomaterials

Ceramics, such as hydroxyapatite (HA), tricalcium phosphate (TCP), and bioglass, are widely used in bone tissue engineering due to their tunable bioactivity. Some of these biomaterials closely resemble the mineral composition of bone and can directly stimulate bone formation.^[^ ^34^ ^]^ For example, HA is biodegradable, chemically biocompatible, and therefore exhibits high osteoconductivity. It dissolves in vivo, releasing calcium and phosphate ions that promote osteoblast activity.^[^ ^3^ ^]^ Bioglass, composed of SiO_2_, P_2_O_5_, and CaO, forms a calcium phosphate layer upon contact with biological fluids, enhancing osteogenesis. However, its mechanical properties differ from natural bone, limiting its use in load‐bearing applications.^[^ ^3^, ^63^ ^]^

While ceramics are ideal for compressive load‐bearing applications, their brittleness and low tensile strength are significant drawbacks.^[^ ^61^ ^]^ These limitations can be mitigated by combining ceramics with polymers to form composite scaffolds.

##### Polymeric Biomaterials

Polymers, both synthetic and natural, are widely used due to their tunable properties. Examples of synthetic polymers include polycaprolactone (PCL), polylactic acid (PLA), and polyglycolic acid (PGA). These materials offer precise control over degradation rates, porosity, and mechanical properties, making them suitable for large‐scale production. However, synthetic polymers lack bioactivity, and their acidic degradation products can cause localized inflammation.^[^ ^15^, ^64^ ^]^ Examples of natural polymers are chitosan,^[^ ^65^ ^]^ gelatin,^[^ ^47^ ^]^ and silk fibroin,^[^ ^66^ ^]^ which are commonly used due to their excellent chemical biocompatibility and cell adhesion properties. However, challenges such as variability in sourcing and difficulty in tuning mechanical properties limit their scalability.^[^ ^66^, ^67^, ^68^ ^]^ PCL is a popular polymer for bone tissue engineering due to its long degradation time and ability to maintain structural integrity in biological environments. When combined with ceramics or MPs, PCL scaffolds exhibit enhanced mechanical properties and support osteogenic differentiation.^[^ ^42^ ^]^

##### Composite Biomaterials

Composite biomaterials merge polymers with ceramics, combining the best of both worlds. They exhibit favorable attributes for bone tissue engineering, including enhanced mechanical toughness, improved chemical biocompatibility, reduced susceptibility to creep‐induced failure, effective load‐bearing capabilities, and bioactivity.^[^ ^67^ ^]^ Some of the most notable composite biomaterials in the literature combine ceramics with polymers.^[^ ^9^, ^12^, ^46^ ^]^ Other literature reviews can be consulted for additional examples of magnetic composite biomaterials used in bone tissue engineering.^[^ ^3^ ^]^

The incorporation of MPs into biomaterials has emerged as a promising strategy as a composite biomaterial to enhance both the mechanical performance and biological functionality of scaffolds used in bone tissue engineering. By tuning particle concentration and scaffold composition, researchers have demonstrated improvements in mechanical strength, surface properties, and cellular responses, although excessive MP loading can introduce structural limitations. Generally, MSs can be created by either physical adsorption by immersing the produced scaffolds in a solution containing the MPs^[^ ^68^ ^]^ or by introducing the MPs to the feedstock during the scaffold manufacturing process,^[^ ^59^ ^]^ with the latter being the most commonly used approach in the literature.

The addition of MPs has consistently been shown to improve the mechanical integrity of scaffolds. For instance, Sahmani et al. investigated scaffolds composed of HA coated with gelatin‐ibuprofen and integrated with increasing concentrations of magnetite MPs.^[^ ^16^ ^]^ They reported that raising the MP content from 0 to 15 wt.% resulted in substantial mechanical enhancements: compressive strength increased by 50%, fracture toughness doubled, elastic modulus increased by 65% and hardness improved by 55%. However, this reinforcement was accompanied by a 20% reduction in porosity, which may influence nutrient diffusion and cell infiltration. Similar mechanical benefits were observed by Kim et al., who incorporated magnetite MPs into PCL scaffolds.^[^ ^69^ ^]^ Their results confirm the general trend that MP integration contributes to increased stiffness and load‐bearing capacity, key requirements for orthopedic scaffold applications.

While moderate MP inclusion improves scaffold properties, exceeding optimal concentrations can negatively impact mechanical performance. Gloria et al. demonstrated that PCL scaffolds loaded with iron‐doped hydroxyapatite (FeHA) particles beyond 20 wt.% showed a decline in mechanical strength.^[^ ^9^ ^]^ This reduction was attributed to the formation of “weak points” within the scaffold matrix, caused by stress discontinuities and poor stress transfer at the particle–polymer interface. Interestingly, despite the mechanical drawback, higher FeHA content enhanced scaffold hydrophilicity, a surface property that correlated with improved cell attachment. Furthermore, scaffolds containing 20 wt.% FeHA (PCL/FeHA 80:20) showed a 20% increase in osteoblast proliferation and ALP expression compared to PCL‐only scaffolds after 14 days in osteogenic media. This finding suggests that MP‐induced changes in surface chemistry and wettability may offset some of the mechanical disadvantages by promoting osteogenic activity.

Beyond direct effects on osteoblast behavior, MPs may also modulate intercellular signaling within the bone microenvironment. Yue Zhu et al. employed a transwell co‐culture system to examine the interaction between osteoblasts and osteoclasts in the presence of MP‐integrated HA scaffolds.^[^ ^70^ ^]^ Their results indicated that MPs influenced osteoblast proliferation indirectly, by altering the composition of osteoclast‐derived exosomes. This suggests a broader regulatory role for MPs in bone remodeling, extending beyond scaffold‐cell interactions to affect paracrine communication between cell types.

#### 3.4.2 Production Methods of Magnetized Scaffolds

The production of MSs plays a crucial role in advancing bone tissue engineering by enabling localized magnetic stimulation and enhancing scaffold performance. A key design consideration is achieving a high magnetic response, typically indicated by a large magnetization value of the incorporated MPs. This allows scaffolds to be manipulated by low‐intensity external magnetic fields, reducing the required MP concentration and thereby minimizing associated toxicity risks.^[^ ^7^ ^]^

**Figure 6:**
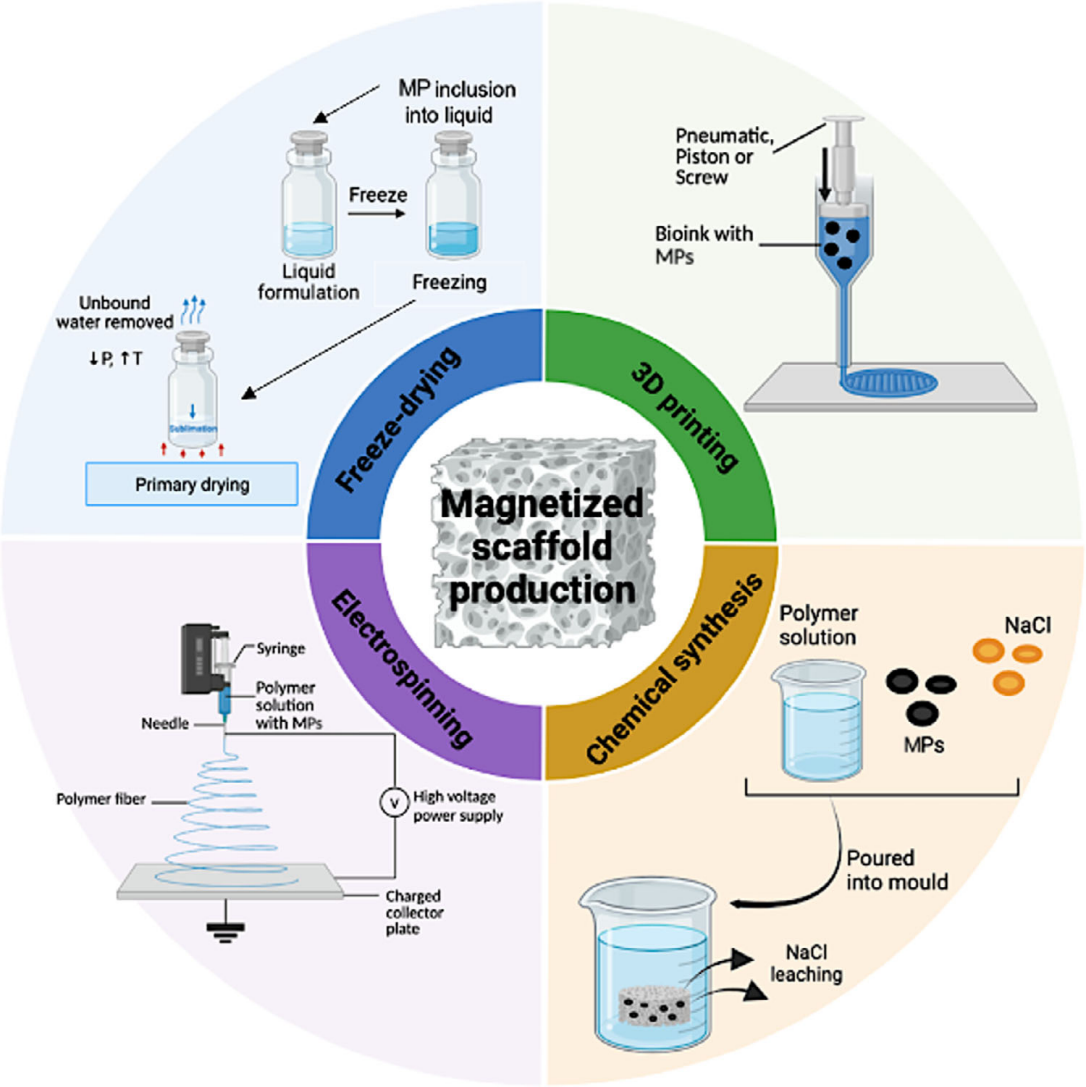
Overview of MS production methods, including freeze‐drying, electrospinning, 3D printing, and chemical synthesis.

**Figure 7:**
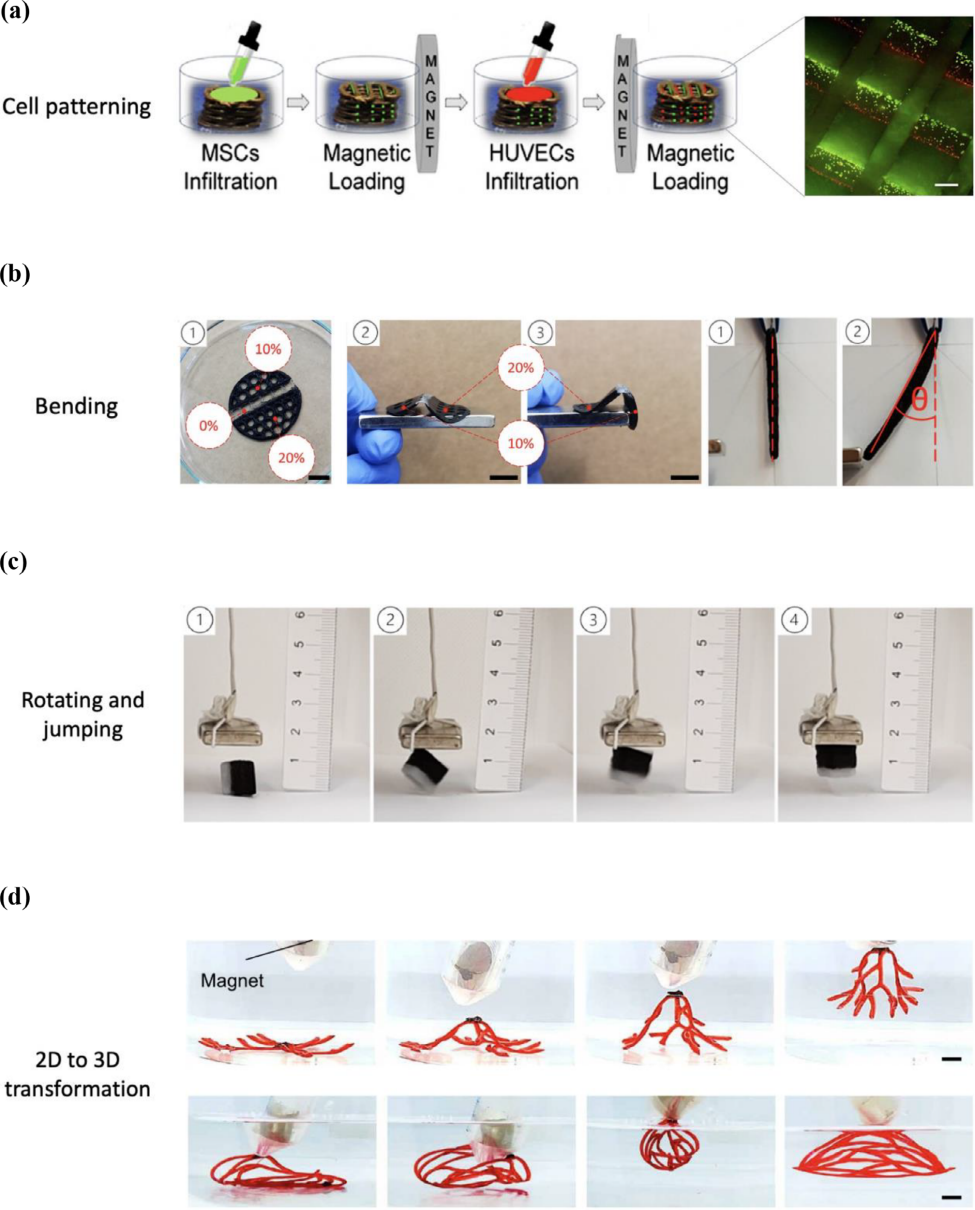
Configuration for assembling magnetically labeled cells for a) cell patterning within the MS with MSCs (green) and HUVECs (red). Scale bar: 200 µm (images adapted with permission.^[^ ^85^ ^]^ Copyright, 2020 Springer Nature); soft robotics created for b) bending, c) rotating and jumping up to 5 mm due to an applied magnetic field (images adapted with permission,^[^ ^86^ ^]^ Copyright 2022, Elsevier), and d) 2D–3D transformation of scaffolds (top row) in air and (bottom row) underwater immersion (images adapted with permission.^[^ ^87^ ^]^ Copyright 2024, Science Advances).

Several fabrication techniques have been employed to integrate MPs into scaffold matrices, each offering distinct advantages in controlling scaffold architecture, porosity, mechanical strength, and magnetic behavior. The most widely applied methods include freeze‐drying, electrospinning, 3D (bio)printing, and chemical synthesis (**Figure** **6**). This section highlights representative studies utilizing each approach, as summarized in **Figure** **7** and **Table** **4**.

**Table 4:** Summary of MS fabrication techniques for bone tissue engineering.^[^ ^93^ ^]^

Method	Benefits	Drawbacks
Freeze‐drying	Does not require leaching steps; no high temperatures; high porosity and interconnectivity; control of pore size	Small pore size; high energy use; use of cytotoxic solvents; time‐consuming
Electrospinning	Simple process; easy to scale up for mass production; high aspect ratio, surface area, permeability, porosity; and surface modification	Limited mechanical properties; organic solvents can be toxic; requires high voltage; poor pore size and shape control; inability to create complex 3D structures
3D printing	Control over geometry and porosity; reproducibility; and tunable mechanical properties	Slow process: post‐processing may be required; prone to clogging; resolution depends on the type of method and machine used
Chemical synthesis	Relatively simple process; cost‐effective; suitable for various polymers	Pore size and distribution are less controllable in the salt leaching method. Low mechanical properties. Possibility of residual solvent and space‐holder/salt particles.

*Freeze‐drying* is a conventional method used to fabricate porous ceramic or composite scaffolds. It involves rapid freezing of the material, followed by sublimation of the solvent, which results in scaffolds with a highly porous, columnar structure.^[^ ^6^ ^]^ When MPs are included, applying an external magnetic field during freezing can orient MP chains along the field lines, improving magnetic anisotropy within the scaffold.^[^ ^71^ ^]^

In a study by Govindan et al., chitosan‐gelatin/phosphate glass scaffolds were prepared with increasing concentrations of magnetite (0–1.5 wt.%).^[^ ^72^ ^]^ The scaffolds exhibited interconnected pores (20–150 µm), and MPs inclusion led to several notable effects: surface roughness increased with MPs concentration, potentially improving cell adhesion, porosity decreased from ≈85% to ≈65%, likely due to MP‐induced densification, swelling capacity was halved, attributed to electrostatic interactions between MPs and polymer carboxylate groups, degradation rate was also reduced, possibly due to diminished hydrophilicity, and compressive modulus increased fourfold at 1.5 wt.% MPs, indicating significant mechanical reinforcement.

These findings demonstrate the ability of freeze‐drying to produce mechanically stable MSs with tunable physical properties through MPs integration.

*Electrospinning* is a widely used technique that produces nanofibrous scaffolds by applying high voltage to a polymer solution, generating fibers that are collected on a charged surface. These nanofibers closely mimic the ECM, making them highly favorable for bone tissue engineering.^[^ ^6^, ^73^ ^]^

Khalili et al. developed electrospun PCL scaffolds incorporating 0.1% (w/v) dendrimer‐modified magnetic particles (mean size: ≈18 nm).^[^ ^42^ ^]^ Using optimized parameters, 20 kV voltage, 0.3 mL h^−1^ flow rate, and 20 cm collector distance, they achieved a significant reduction in fiber diameter in MSs (495 ± 144 nm) compared to non‐magnetized controls (866 ± 310 nm), likely due to increased solution conductivity. This also led to improved fiber uniformity and enhanced structural homogeneity. Similarly, Chen et al. incorporated Fe_2_O_3_ MPs coated with PSC into poly(lactic‐co‐glycolic acid) PLGA and PCL scaffolds, fabricated under 16 kV and 0.8 mL h^−1^ flow rate.^[^ ^48^ ^]^ Their scaffolds exhibited fiber diameters ranging from 0.7 to 1.4 µm and demonstrated a higher Young's modulus (1.25 GPa for magnetized scaffolds vs 0.75 GPa for controls), confirming improved stiffness. However, the softer PSC coatings slightly reduced mechanical strength compared to thinner‐coated nanoparticles like citrate‐gold.

In Yu et al., electrospinning played a central role in fabricating nanofibrous scaffolds composed of PLGA integrated with iron‐doped hydroxyapatite (Fe‐HA) nanoparticles.^[^ ^74^ ^]^ This method enabled the creation of a porous network structure, ideal for bone tissue engineering, as confirmed by scanning electron microscopy. The Fe‐HA nanoparticles, characterized as needle‐like crystals, were uniformly distributed within the PLGA matrix, preserving their superparamagnetic properties. This magnetic behavior was crucial for enhancing osteogenic differentiation when the scaffolds were exposed to a static magnetic field. Rat bone mesenchymal stem cells exhibited strong adhesion and proliferation on the electrospun scaffolds, and under magnetic stimulation, they penetrated and grew within the fibrous matrix. The synergistic effect of the magnetic field and scaffold composition led to increased alkaline phosphatase activity and upregulation of osteogenic markers. As further support, Yu et al. demonstrated that combining electrospun PCL/gelatin nanofibers with 3D‐printed PCL meshes yielded composite scaffolds with enhanced porosity (79.32 ± 8.32%) and significantly improved compressive modulus (30.50 ± 0.82 MPa) compared to electrospun‐only scaffolds (18.55 ± 0.56 MPa), confirming the mechanical and biological benefits of hybrid fabrication approaches.^[^ ^75^ ^]^ This positions electrospinning not only as a fabrication method but as a strategic platform for engineering next‐generation scaffolds in regenerative medicine.

*3D (bio)printing* offers unmatched precision in scaffold architecture, allowing the fabrication of highly reproducible, customizable structures with defined porosity and geometry. Based on the material system, methods include: binder jetting / selective laser sintering (SLS) (powder‐based), direct ink writing (DIW) / robocasting / vat photopolymerization (resin‐based), and fused deposition modeling (FDM) (thermoplastic‐based).^[^ ^14^, ^76^ ^]^

Tavares et al. used extrusion‐based 3D printing to fabricate HA‐chitosan‐PVA scaffolds with MPs concentrations of 1.92–5.54 wt.%.^[^ ^77^ ^]^ Their findings included: filament diameter stability, attributed to MP‐chitosan interactions improving shape retention, increased elastic modulus (from 27 ± 8 to 92 ± 4 kPa), demonstrating effective reinforcement, reduced swelling ratio (≈20% decrease), likely due to electrostatic interference with water absorption, and no significant erosion, confirming good structural stability. Other studies have explored various 3D printing routes: Kao et al. employed FDM using PCL, calcium silicate, and magnetite MPs.^[^ ^78^ ^]^ Ngadiman et al. used PLA/maghemite mixed with a bioresin via digital light processing (DLP).^[^ ^62^ ^]^ Yang et al. created dual‐phase scaffolds combining PCL frameworks and MP‐loaded hydrogels (OSA/CMCS).^[^ ^79^ ^]^ These results support the versatility of 3D printing for fabricating MSs with controlled porosity, mechanical reinforcement, and magneto‐responsive behavior.

*Chemical synthesis* offers a versatile platform for engineering MSs through techniques such as cross‐linking, salt leaching, or the space‐holder method, allowing the creation of hydrogels or solid scaffolds with integrated MPs.^[^ ^7^, ^15^, ^29^, ^36^, ^46^, ^47^ ^]^ For instance, Kim et al. used salt leaching to fabricate porous PCL‐based MSs with 0–10 wt.% MPs.^[^ ^69^ ^]^ Their scaffolds achieved: high porosity (65–75%), and enhanced elastic modulus, increasing from ≈1 to 2.5 MPa with MP inclusion. Filippi et al. fabricated PEG‐based magnetized hydrogels by co‐assembling PEG‐coated MPs (15 nm), hydrogel matrices, and human adipose‐derived cells.^[^ ^46^ ^]^ Cross‐linking was achieved enzymatically via Factor XIIIa, resulting in improved mechanical performance, with elastic modulus increasing from 1.72 kPa (cells only) to 3.69 kPa (cells + MPs), and slower stress relaxation, suggesting increased structural stability. Building on these approaches, Samal et al. introduced a multilayered magnetic gelatin membrane scaffold fabricated through chemical cross‐linking and nanoparticle integration.^[^ ^47^ ^]^ By assembling gelatin membranes with varying MP concentrations, they achieved tunable magnetic gradients capable of spatially directing magnetized stem cells under external magnetic fields. Moreover, the scaffolds exhibited localized thermal responsiveness, reaching up to 43.7 °C under oscillating magnetic fields, enabling magnetic hyperthermia and thermal gradient formation within 3D constructs. This strategy exemplifies how chemical synthesis not only supports structural customization but also enables multifunctional magneto‐mechanical behavior such as thermal activation and cell guidance critical for advanced tissue engineering applications.

Each of the above‐mentioned fabrication techniques presents distinct advantages for the integration of MPs into scaffolds: freeze‐drying allows the alignment of MPs and creates porous structures ideal for mineralized tissues; electrospinning produces nanofibrous architectures resembling ECM, supporting cell adhesion and proliferation; 3D printing enables architectural control and structural customization, and chemical synthesis provides routes for incorporating MPs into soft or porous matrices. Selection of the appropriate method depends on the intended application and the associated biocompatibility requirements, such as an optimum mechanical strength and the desired level of magnetic responsiveness.

#### 3.4.3 Influence of Scaffold Magnetization on Bone Cells

In bone tissue engineering, an ideal scaffold should not only provide structural support and promote cell migration but also stimulate cellular proliferation and differentiation. MSs, incorporating MPs, have demonstrated potential in promoting these biological responses, even in the absence of an external magnetic field.

The presence of MPs in the scaffolds appears to initiate molecular and cellular interactions that may influence cell behavior. For example, Tavares et al. reported that MPs incorporated into scaffolds facilitated the binding of vitronectin, a glycoprotein essential for bone cell adhesion.^[^ ^77^ ^]^ This likely promoted the engagement of integrin receptors on cell membranes, leading to downstream activation of signaling pathways responsible for proliferation and osteogenesis.

Supporting this, Yun et al. showed that MSs alone were able to activate integrin‐associated signaling molecules, such as phosphorylated focal adhesion kinase (p‐FAK), p‐paxillin, and RhoA, all of which are crucial in mechanotransduction and cytoskeletal organization.^[^ ^29^ ^]^ These effects were significantly enhanced when MSs were exposed to external magnetic fields (see Section 3.4.4).

Several studies also reported increased cell proliferation and differentiation when using MSs. Nevertheless, while enhanced osteogenic activity was often observed with MSs, Tavares et al. noted that increasing MPs concentration did not consistently correlate with alkaline phosphatase (ALP) expression, suggesting an optimal MPs dosage for a maximum ALP activity.^[^ ^77^ ^]^

#### 3.4.4 Influence of Scaffold Magnetization Combined with Magnetic Field Exposure

When MSs are combined with external magnetic fields, particularly SMFs or AMFs, a synergistic effect often emerges, leading to amplified cellular responses. These combined systems have been shown to stimulate cell adhesion, migration, osteogenic differentiation, and even angiogenesis.^[^ ^29^, ^38^, ^46^, ^78^ ^]^ A constant field of uniform strength was commonly used to investigate baseline changes in cell behavior,^[^ ^78^ ^]^ whereas a time‐varying magnetic field was used to provide cyclic mechanical stimulation, mimicking physiological loading conditions.^[^ ^61^, ^80^ ^]^

In a pivotal study, Yun et al. exposed MSs and non‐magnetized scaffolds to a 15 mT SMF and evaluated osteogenic markers in cultured osteoblasts.^[^ ^29^ ^]^ They observed that the expression levels of RUNX2 and Osterix (early osteogenic transcription factors) increased significantly in MSs under SMF, and ALP activity and calcium deposition were markedly enhanced, particularly by day 10, in magnetized scaffolds compared to controls. Similarly, Khalili et al. demonstrated that applying a pulsed electromagnetic field (PEMF) of 30 mT at 75 Hz for 8 h day^−1^ enhanced osteogenic differentiation in adipose‐derived stem cells. This was evidenced by elevated expression of osteocalcin, collagen type I, RUNX2, and increased ALP activity and mineralization.^[^ ^42^ ^]^ Yun et al. also investigated the underlying signaling pathways involved in these magnetically induced effects.^[^ ^29^ ^]^ They found that MSs combined with SMFs activated integrin signaling (p‐FAK, p‐paxillin, RhoA), BMP2/Smads axis, MAPK, and NF‐κB pathways, key regulators of osteogenesis. In a related study, Zhu et al. reported that MSs promoted osteoblast proliferation through activation of the MAPK/ERK pathway. They proposed that MP‐loaded scaffolds adsorbed specific extracellular proteins that then engaged receptor‐mediated intracellular cascades.^[^ ^68^ ^]^ Notably, across several studies, increasing MPs concentration generally resulted in greater osteogenic stimulation, suggesting the creation of a local microenvironment enriched in nanoscale magnetic domains. These localized fields likely serve as mechanical or electrochemical stimuli, promoting cellular mechanotransduction.^[^ ^68^ ^]^

In addition to promoting osteogenesis, magnetic scaffolds and SMF exposure have also been implicated in vascular tissue development, a critical component of bone regeneration. For instance, Yun et al. demonstrated that stromal vascular fraction (SVF) cells cultured on MSs under a 15 mT SMF exhibited increased expression of vascular endothelial growth factor (VEGF) and angiogenin‐1, as well as capillary‐like tube formation.^[^ ^29^ ^]^ Filippi et al. confirmed these effects using PEG‐based hydrogels embedded with MPs and human adipose cells. Magnetic stimulation significantly upregulated angiogenic markers such as VEGF‐A, CD31, T‐cadherin, VCAM‐1, and α‐SMA.^[^ ^46^ ^]^ These findings point to the dual role of MSs under magnetic field stimulation in promoting both osteogenesis and vasculogenesis, making them particularly promising for treating large or critical‐sized bone defects that require simultaneous bone and vessel formation.

### 3.5 Combined Strategies and Applications Using MPs

A promising direction in bone tissue engineering involves combining magnetized scaffolds and cells to generate remote, non‐contact mechanical stimulation using externally applied magnetic fields. Unlike magnetic hyperthermia, which relies on heat generation, these strategies employ magnetic forces to create mechanical deformation within scaffolds, an approach more suited to supporting bone regeneration without causing local temperature increases.^[^ ^80^, ^81^, ^82^ ^]^ Bone tissue is highly responsive to mechanical cues, and mechanical loading plays a vital role in regulating osteoblast activity and bone remodeling.^[^ ^8^ ^]^ Specifically, mechanical stimulation has been shown to suppress sclerostin expression, a protein secreted by osteocytes that inhibits osteogenesis. Its downregulation activates Wnt signaling, promoting osteoblast differentiation and enhancing bone formation.^[^ ^83^ ^]^

#### 3.5.1 Integration Strategies

Two main approaches have emerged in using MPs in bone tissue engineering: scaffold magnetization only, where MPs are incorporated into scaffold matrices and non‐magnetized cells are seeded, and dual magnetization, where both the scaffold and the cells are magnetized using MPs. The impact of these strategies has been systematically reviewed in **Tables** **5** and **6**, which categorize studies based on scaffold fabrication method, materials, MPs content, magnetic characterization, stimulation type, and biological outcomes. **Table** **7** summarizes studies employing the combined approach, where both cells and scaffolds are magnetized.

**Table 5:** Summary of selected articles using magnetized scaffolds seeded with non‐magnetized cells under no externally applied magnetic field.

Materials	Scaffold Fabrication Method	MP Synthesis Method	Mp Content	Saturation Magnetization	Key Findings	Ref.
Iron oxide MPs, HA	Space holder technique	Co‐precipitation	0, 5, 10, 15 wt.%	‐	Compressive strength, fracture toughness, elastic modulus, and hardness increased as MPs concentration increased while the porosity decreased.	[16]
Magnetite MPs (15–30 nm), PCL/Col1 nanocomposite scaffold, ADSCs	Electrospinning	Co‐precipitation	5 wt.%	65 emu g^−1^	MPs enhanced scaffold properties (strength, wettability, and porosity); promoted ALP activity and calcium mineralization; upregulated osteogenic‐related genes or proteins (Col1, RUNX2, OCN, OPN, BMP2).	[12]
Magnetite MPs, HA, mouse macrophages	Purchased	Hydrothermal	‐	‐	Intrinsic magnetism in the scaffold activates PPAR signal transduction in macrophages, resulting in downregulated JAK‐STAT signals and M2 macrophage polarization.	[30]
Iron oxide MPs, HA and PLGA bioink, embryonic murine C3H10T12 cells, and human‐patient‐derived osteoblast‐like HBO cells	Extrusion‐based 3D printing	‐	60, 200 µg mL^−1^	‐	Viable and functional in vitro culture of cells up to 14 days on printed scaffolds. MPs addition reduced the activity of *Staphylococcus aureus*.	[37]
Maghemite MP (9 nm), calcium phosphate, chitosan solution, human dental pulp stem cells	In situ precipitation	Co‐precipitation	0% 1% 3% 6%	0 emu g^−1^ 0.1 emu g^−1^ 0.35 emu g^−1^ 0.8 emu g^−1^	MPs promoted osteogenesis by hDPSCs, ALP activity, expression of osteogenic genes, and bone matrix formation, with the 3% MPs showing the best results. The osteogenic behavior of hDPSCs was likely driven by CPC+IONP via the WNT signaling pathway.	[52]
PLA‐Maghemite MPs (8–13 nm), Ultra‐high and flexible bioresin, human skin fibroblast cells	Digital light processing (3D printing)	Co‐precipitation	1 v/v% 3 v/v% 5 v/v%	‐	PLA alone and PLA‐MPs elevated the scaffold's Young's modulus. While PLA alone decreased cell viability, PLA‐MPs enhanced it, though both remained lower than the control, which had no PLA or PLA‐MPs.	[62]
Iron oxide MPs (10 nm), PCL, MC3T3‐E1 preosteoblasts	Salt‐leaching method	Thermal decomposition	0 wt.% 5 wt.% 10 wt.% 100 wt.%	0 emu g^−1^ 1.6 emu g^−1^ 3.1 emu g^−1^ 72.1 emu g^−1^	Improved hydrophilicity, water swelling, mineral induction, cell adhesion, and mechanical stiffness in magnetic scaffolds.	[69]
Magnetite MPs, HA, MC3T3‐E1 preosteoblasts	Foamed‐based and microwave‐assisted heating	Thermal decomposition	10 wt.% 100 wt.%	2 emu g^−1^ 56 emu g^−1^	Magnetized scaffolds increased cell proliferation through the MAPK/ERK cascades.	[68]
Magnetite MPs (50 nm), PCL, MC3T3‐E1 pre‐osteoblasts	Thermally Induced Phase Separation, then freeze‐drying	Supplied by Sigma–Aldrich	5 wt.% 10 wt.% 15 wt.% 20 wt.% 100 wt.%	78 emu g^−1^ 81 emu g^−1^ 75 emu g^−1^ 69 emu g^−1^ 82 emu g^−1^	PCL was non‐cytotoxic for 72 h, but scaffolds with 20% MPs approached cytotoxic limits. Incorporating MPs into PCL altered its surface from hydrophobic to hydrophilic.	[64]
Magnetite MPs (two batches of *<* 5* µm* or *<* 50* nm*), PCL, human fibroblast 3T3 cells	Salt leaching technique	‐	0, 5, 10, 20, 40 w/w% of *<* 5* µm* and 5, 10, 20, 40 w/w% of *<* 50*nm*	21.09 emu g^−1^ for 20w/w% *<* 5* µm;* 1.18 emu g^−1^ for 20w/w% *<* 50* nm*	MP to PCL wt ratio of 0.1:0.9 showed superior mechanical properties (Young's modulus: 1 MPa, stiffness: 13 N mm^−1^). Micron‐sized MPs had cell viability below 80%, while nano‐sized MPs were all above 80%.	[15]
Magnetite MPs, phosphate glass, chitosan, human osteoblast‐like MG‐63	Freeze drying technique	Co‐precipitation	0.5wt.% 1 wt.% 1.5 wt.%	0.180 emu g^−1^ 0.432 emu g^−1^ 0.496 emu g^−1^	MP‐polymer interactions reduced scaffold swelling and weight loss by decreasing hydrophilicity. The stress–strain curve showed improved mechanical properties in CG/PG/MP scaffolds; they didn't fracture up to 90% strain. Cell viability exceeded 80%, decreasing slightly with 1.5% MPs.	[72]
Aminated iron oxide MPs (11 nm), oxidized sodium alginate / carboxymethyl chitosan hydrogel, PCL, bone marrow mesenchymal stromal cells	Hydrogel‐integrated 3D printed scaffold	Co‐precipitation	3, 6, 12, 24, 48 µg mL^−1^ in scaffold	‐	The hydrogel carried drugs; adding simvastatin boosted osteogenic markers in the scaffold.	[79]
Magnetite MPs, HA, chitosan, cellulose fibers, MC3T3‐E1 preosteoblasts	Freeze‐drying under a magnetic field	Co‐precipitation	‐	‐	Magnetized scaffolds increased cell proliferation and osteogenic differentiation but inhibited osteoclastic differentiation. The tensile and compressive strength of the magnetized scaffold increased along the perpendicular direction to the fibers.	[94]

**Table 6:** Summary of selected articles using magnetized scaffolds seeded with non‐magnetized cells under an externally applied magnetic field.

Materials	Scaffold Fabrication Method	MP Synthesis Method	MP Content	Saturation Magnetization	Stimulation Type And intensity	Key Findings	Ref.
Iron oxide MPs, HA + chitosan + PVA polymeric matrix, human osteosarcoma Saos‐2 cells	Extrusion‐based 3D printing	Co‐precipitation	1.92 wt.% 3.77 wt.% 5.54 wt.%	‐	Magnetic flux density of 300 gauss and a frequency of 418.5 kHz, for 10 min	Scaffolds with 3.77% and 5.54% MPs achieved 6.6 and 7.5 °C temperature rises in hyperthermia testing. Superparamagnetic MPs significantly promoted cell adhesion, proliferation, and ALP expression.	[77]
Iron‐doped hydroxyapatite MPs (FeHA) (13 nm), PCL matrix, hMSCs	Moulding and solvent‐casting	Protocol by Tampieri A et al.^[^ ^95^ ^]^	Polymer‐to‐particle weight ratios of 90/10w/w 80/20w/w 70/30w/w	0.9 emu g^−1^ 0.6 emu g^−1^ 0.3 emu g^−1^	Oscillating magnetic field (f = 260 kHz and 27 mT amplitude)	PCL/FeHA 90/10 shows higher mechanical performance. Increased MPs weaken the scaffold, lower mechanical performance. MPs enhance hydrophilicity. More Fe leads to higher hyperthermia temperatures. Better cell attachment and proliferation in PCL/FeHA correlate with water contact angle.	[9]
Magnetite MPs (10 nm), PCL, primary culture of mouse calvarial osteoblasts	Freeze‐drying + salt leaching method	Thermal decomposition	5 wt.% 10 wt.%	1.7 emu g^−1^ 4.8 emu g^−1^	SMF, 15mT	Osteoblast differentiation, bone formation, and activation of endothelial cell angiogenesis in the SMF + magnetized scaffolds. BMP and integrin‐pathways involved in SMF and magnetic scaffold induced osteoblast differentiation.	[29]
Iron oxide MP (10 nm), decellularized ECM/regenerated silk fibroin, rat BMSCs	Soaking the decellularized ECM in the precursor solution	‐	3 wt.% 6 wt.%	0.86 emu g^−1^ 2.2 emu g^−1^	SMF, 120–130 mT	Accelerate new bone formation and angiogenesis. SMF enhanced the chemotaxis effect. Effect on osteogenesis was mediated by the intracellular *Ca* ^2+^/CaM/CaMKII signaling axis, which was initiated by ER *Ca* ^2+^ release via upregulation of CASQ2.	[38]
Dendrimer‐modified iron oxide MPs (18 nm), PCL nanofibers, ADMSCs	Electrospinning	Co‐precipitation	0.1% (w/v)	57.75 emu g^−1^ for pure MPs	Pulsed EMF exposure at 30 mT, 75 Hz, 8 h/day	MP incorporation reduced nanofiber size, enhanced cell attachment, and growth. ADMSCs on MP‐PCL with osteogenic media and PEMF exposure exhibited elevated Osteocalcin, RUNX2, calcium content, and ALP activity.	[42]
PEG‐functionalized iron oxide (II, III) MPs (15 nm), PEG hydrogel, and Stromal vascular fraction cells	Hydrogel polymerization – chemical crosslinking	Purchased from Sigma–Aldrich	500 µg Fe/g gel	‐	SMF, 50 mT	Enhance endothelial proliferation for vascular development and promote the deposition of a calcified matrix through osteogenic differentiation in vitro.	[46]
Magnetite MPs (100 nm), PCL, calcium‐Silicate, Wharton's jelly mesenchymal stem cells	3D printing (fused deposition modeling)	Purchased from Sigma–Aldrich	0 wt.% 2.5 wt.% 5 wt.%	‐	SMF, 20 min daily	5% Fe3O4 yielded optimal compressive strength (9.6 ± 0.9 MPa) and degradation rate (21.6 ± 1.9% in four weeks). The Fe3O4‐containing scaffold enhanced in vitro bioactivity, differentiation, and stem cell adhesion. Under SMF, the CS scaffold with Fe3O4 not only boosted cell activity but also prompted simultaneous secretion of collagen I and osteocalcin.	[78]
Maghemite MPs (7–8 nm), non‐magnetic iron oxide nanoparticles, calcium phosphate cement (CPC), human dental pulp stem cells	Injectable scaffold fabrication (powder‐forming processes)	Co‐precipitation	24 mg mL^−1^, pure MPs	0.6 emu g^−1^ 80 emu g^−1^	SMF, 35 ± 5 mT	Under SMF, MP‐CPC enhanced hDPSCs’ performance, exhibiting 3 fold higher alkaline phosphatase activities, increased expressions of osteogenic marker genes, and enhanced cell‐synthesized bone minerals. TEM revealed nanoaggregates within cells, suggesting improved performance due to both the magnetic field's physical forces and cell internalization of released MPs from MP‐CPC constructs.	[59]
Zr–Fe3O4, bioactive wollastonite, and calcium silicate bioglass	Space holder method	Purchased from Merck Company, Germany	0 wt.% 5 wt.% 10 wt.% 15 wt.%	4 emu g^−1^ 9 emu g^−1^ 15 emu g^−1^ 50 emu g^−1^	AC field at 100–250 kHz	Scaffolds with 10 and 15 wt.% MPs had better biological and mechanical responses. By increasing the volume of MP content, the porosity decreased, and compressive strength increased.	[61]
Dextran‐grafted maghemite MPs, Chitosan with L‐Arg as cross‐linker agent, Undifferentiated human mesenchymal stem cells	Freeze‐drying	‐	5%–10%, 15% w/w	‐	AMF 70 Hz and 25–30 mT, for 6 h per day (20 intervals–18 min each)	Cell metabolic activity increases in the presence of L‐Arg. Magnetic stimulation did not have any significant changes compared to non‐stimulated scaffolds (a slight decrease was observed).	[65]
Iron (II, III) oxide nanoparticle (50–100 nm), chitosan hydrogel, 7F2 osteoblast cells	Extrusion based 3D printing	Bought from sigma	0, 0.66, 1.33, 2.00, 2.66, and 3.33 mg mL^−1^	‐	PEMF 3.0 mA, 30 V, pulse frequency = 75 Hz, pulse width = 1.3 ms for 2 h/day	Modified MPs, less prone to agglomeration, were retained in positively charged chitosan. Magnetic chitosan scaffolds induced a coupling force, enhancing bone cell growth and mineralization (inductive coupling resulted in increased calcium secretion).	[96]
HA‐Magnetite MPs (5–20 nm in width and up to 50–80 nm in length), PLGA, rat bone mesenchymal stem cells	Electrospinning	Co‐precipitation	‐	7.61 emu g^−1^ for pure MPs and 1.19 emu g^−1^ after incorporation to scaffold	SMF with the strength of 300 Gs	Needle‐like MPs facilitate cell penetration into PLGA/Fe‐HA scaffolds under a static magnetic field. Elevated alkaline phosphatase activity and osteogenic factor expression indicated induced osteoblastic differentiation in mesenchymal stem cells through the combined influence of the magnetic scaffold and static magnetic field.	[74]
HA doped magnetite MPs, collagen, MG63 human osteoblast‐like cells	Freeze‐drying	Co‐precipitation	6.2 wt.%	0.091, 0.182, and 1.103 emu g^−1^ or 25, 40, and 50 °C composite preparation temperatures	SMF, 320 mT	Composite synthesis at higher temperatures reduced degradation/weight loss. Scaffold synthesized at 25 °C, with applied SMF, exhibited the highest cell proliferation. Gene expression analysis revealed cell differentiation induced by both MP inclusion and SMF.	[97]

**Table 7:** Summary of selected articles using magnetized scaffolds seeded with magnetized cells under an externally applied magnetic field.

Materials	Scaffold Fabrication Method	MP Synthesis Method	MP Content	Saturation magnetization	Stimulation Type and Intensity	Key Findings	Ref.
(PAA)‐coated iron MPs (9 nm), gelatin protein network, hMSC	Gelation of membranes, cast into plates and then cross‐linked	Alkaline co‐precipitation	In scaffold: MP ratios of 0, 1.7, 3.4, 6.7, 16.7, 33.4, 50 66.7, 83.4 (w/v%). In cells: 50, 100, and 200 pg cell^−1^	For in scaffold: 0.3, 0.6, 0.8, 2.6, 6.1, 10.1, 16.1, 38.7 emu g^−1^, respectively	SMF 1.2T on scaffold + cell system	Membranes with higher MPs content are topographically rough but more homogeneous. Increased MPs reduce hydrophilicity due to water evaporation in Differential Scanning Calorimetry. Higher MP concentration enhances temperature and hyperthermia properties, creating temperature gradients in the scaffold.	[47]
Magnetite MPs (50–100 nm), HA, PCL, Mesenchymal Stromal Cells, magnetized NIH/3T3 fibroblast line	Extrusion based 3D‐printing	Purchased from Sigma	0.5 and 1%	‐	SMF, NdFeB permanent magnets (diameter 15 mm, length 5 mm, Br = 1.2 T) distanced 27 mm	1% MPs showed good cell proliferation and intrinsic osteogenic potential, indicating no toxic effects of the employed scaffold materials. Higher adhesion rates with no cytotoxic effects were found in magnetized cells.	[91]

#### 3.5.2 Magnetized Bioinks and Scaffold Mechanics

One innovative avenue has been the development of magnetized bioinks for 3D bioprinting. Spangenberg et al. formulated a magnetic bioink containing 3% alginate, 9% methylcellulose, and 25% w/w magnetite MPs (diameter range: 15–100 µm).^[^ ^80^ ^]^ Their findings indicated that at MP concentrations above 15%, extrusion pressure increased, compromising printability. Shape fidelity improved with MPs content, i.e., strands maintained consistent geometry, approaching an ideal fidelity ratio (≈1.0), compared to a ratio of ≈1.5 in non‐magnetic bioinks. In a filament collapse test, magnetic bioinks were more prone to collapsing over longer spans due to the increased weight, exerting greater pressure on underlying layers. Young's modulus and saturation magnetization increased linearly with MP content, confirming tunable mechanical and magnetic performance. Importantly, cell viability was unaffected, as demonstrated via LDH assay comparing MS extracts to controls, including pure magnetite powder.^[^ ^80^ ^]^ To investigate magnetically induced scaffold deformation, Spangenberg et al. exposed printed scaffolds to a low‐frequency (0.05 Hz) magnetic field with a field gradient of 10 kAm^−1^ × 1 mm^−1^, maximum strength of 160 kA m^−1^, and flux density of 200 mT. Deformation varies with scaffold geometry and orientation, highlighting the importance of structural design in magnetically responsive systems. Building on this work, Czichy et al. developed a magnetic bioreactor tailored to stimulate such scaffolds under optimal cell culture conditions.^[^ ^81^ ^]^ They later proposed a bending model based on Kelvin force and beam theory to predict deformation dynamics in magnetized alginate‐based structures.^[^ ^84^ ^]^

#### 3.5.3 Guided Cell Patterning using MPs

Beyond bulk scaffold stimulation, external magnetic fields can also be used to spatially guide and pattern cells. Goranov et al. magnetically labeled MSCs and human umbilical vein endothelial cells (HUVECs) with MPs, then deposited them on opposite sides of MS fibers using inhomogeneous magnetic gradients (Figure 7a).^[^ ^85^ ^]^ The scaffolds were fabricated via extrusion‐based 3D printing using Fe‐doped HA and PCL. This technique enabled controlled spatial organization of multiple cell types within a single construct, potentially enhancing tissue integration and vascularization.

#### 3.5.4 Smart Magnetic Hydrogels and Soft Robotics Applications

Emerging studies are also exploring magneto‐responsive hydrogels that mimic soft robotic behavior, i.e., capable of shape transformation, actuation, and dynamic deformation, with potential applications in mechanically stimulating cells within bone scaffolds. Siminska–Stanny et al. engineered composite hydrogels with magnetic and non‐magnetic regions. These constructs demonstrated macroscopically anisotropic behavior, capable of bending, rotating, and even jumping in response to magnetic fields.^[^ ^86^ ^]^ The actuation performance was tunable by altering the distribution and interaction of the magnetic zones within the hydrogel (Figure 7b,c). Xie et al. developed sacrificial magnetic ink‐based hydrogels that undergo a 2D‐to‐3D transformation when exposed to a magnetic field. This innovative printing strategy enabled the generation of complex geometries from flat scaffolds, offering new possibilities for programmable, responsive architectures in bone tissue engineering (Figure 7d).^[^ ^87^ ^]^

These soft material systems, while still in the early stages of development for possible applications in regenerative medicine, represent a frontier in non‐invasive mechanical stimulation, potentially mimicking physiological forces such as tension, compression, or bending, all critical in guiding osteogenic differentiation. The integration of MPs into both scaffolds and cells, combined with the application of external magnetic fields, offers a non‐contact, dynamically tunable platform for stimulating bone regeneration. Whether through mechanical actuation, spatial cell patterning, or smart hydrogel deformation, these systems create microenvironments that mimic native mechanical stimuli, enhance osteogenesis, and potentially accelerate healing in complex bone defects. Continued interdisciplinary research (i.e., combining biomaterials science, cell biology, and biomechanics) is essential to further optimize these magnetic systems and translate them toward clinical applications in orthopedics and beyond.

## 4 Discussion

The use of magnetized systems (i.e., MCs, MSs, or a combination of both) represents a promising and increasingly studied approach in bone tissue engineering. MPs, typically based on iron oxides, have been employed to influence cell behavior and scaffold performance through remote, non‐contact mechanical stimulation, modulation of surface properties, and activation of intracellular signaling pathways.

### 4.1 Strategies for Cell and Scaffold Magnetization

Cell magnetization is primarily achieved via MPs uptake by endocytosis, though surface adsorption has also been used. Similarly, scaffold magnetization is typically conducted by either incorporating MPs directly into scaffold base materials or coating prefabricated scaffolds via immersion in MPs suspensions. Of these, bulk incorporation remains the most prevalent due to its improved stability and uniformity.

Most MPs used in the reviewed studies were either commercially available or synthesized via co‐precipitation, a widely adopted method due to its scalability and simplicity. However, MPs agglomeration remains a recurring challenge, especially in extrusion‐based 3D printing, necessitating the use of surface coatings. Despite numerous coating strategies (e.g., PEG, citrate, phosphonates), there is no standardized approach optimized for bone cell applications.

### 4.2 Fabrication Techniques and Printing Considerations

MSs are commonly fabricated using polymers, ceramics, or composites, with scaffold manufacturing methods including freeze‐drying, electrospinning, 3D printing, and chemical synthesis. Among these, extrusion‐based 3D printing is particularly promising due to its control over scaffold geometry and material distribution, including multi‐material capabilities. However, the 3D‐printing of magnetic hydrogels is still in its early stages. Key challenges include: 1) lack of standardization in bioprinting parameters, 2) limited rheological data on magnetized bioinks, 3) unclear effects of MPs agglomeration, particularly during the pre‐print, print, and post‐print phases.

While most studies have focused on alginate‐ or cellulose‐based hydrogels, more research is needed to explore alternative bioinks, investigate their mechanical and flow properties, and assess how MPs influence print fidelity and cell viability, particularly in the presence of an external magnetic field.

### 4.3 Mechanical and Surface Property Enhancements

The inclusion of MPs into scaffolds consistently improves mechanical performance, including compressive strength, fracture toughness, elastic modulus, and hardness. These improvements make MSs particularly suitable for hard tissue applications, such as bone, which typically range in Young's Modulus values of MPa‐GPa. In addition, increased surface roughness has been directly correlated with enhanced cell adhesion, providing more binding sites for focal adhesion complexes.^[^ ^9^, ^16^ ^]^ However, excessive MPs concentrations (usually higher than polymer:MP weight ratios of 80:20) can introduce mechanical vulnerabilities due to stress concentrations at particle‐matrix interfaces.^[^ ^9^ ^]^ Moreover, MPs tend to reduce scaffold porosity, which may impede nutrient diffusion and cell infiltration.

There is currently no standardized framework for magnetized scaffold design, MPs concentration, (bio)ink preparation (in case of 3D (bio)printing), or characterization protocols. Establishing such norms would greatly aid reproducibility and translational potential.

### 4.4 Biological Effects and Magnetic Stimulation

Beyond mechanical reinforcement, MPs play a biological role by modulating cell proliferation and osteogenic differentiation. Mechanistically, internalized MPs can suppress intracellular H_2_O_2_, supporting cell survival,^[^ ^44^ ^]^ MPs can activate the MAPK signaling pathway, promoting osteogenesis,^[^ ^3^ ^]^ and external magnetic fields (i.e., static or alternating) can enhance these effects, particularly by interacting with non‐superparamagnetic MPs (which retain an intrinsic magnetic field).^[^ ^59^, ^78^ ^]^

Magnetic stimulation modalities differ significantly in their underlying biophysical mechanisms. SMFs primarily exert mechanical effects, inducing torque or magnetic forces on internalized or scaffold‐embedded particles. These mechanical cues activate integrin‐based signaling pathways (e.g., focal adhesion kinase, paxillin, RhoA), and in combination with magnetic scaffolds, have been shown to upregulate osteogenic transcription factors and bone morphogenetic proteins, as demonstrated by Yun et al., who observed these effects under a 15 mT SMF in combination with magnetic scaffolds.^[^ ^29^ ^]^ In contrast, PEMFs operate via electromagnetic induction, producing localized electric currents within tissues.^[^ ^88^ ^]^ These microcurrents can alter the resting membrane potential of cells, potentially opening voltage‐gated ion channels, particularly Ca^2^⁺ channels. This influx of calcium ions is a key trigger in second messenger systems such as calmodulin‐dependent cascades, which are essential for regulating cell proliferation, migration, and differentiation.^[^ ^89^ ^]^ These effects have been observed in multiple studies. For example, Khalili et al. demonstrated increased osteogenic differentiation markers of adipose‐derived MSCs exposed to PEMF (30 mT, 75 Hz),^[^ ^42^ ^]^ while Huang et al. observed upregulated type I collagen expression and ALP activity under similar conditions.^[^ ^35^ ^]^ Recognizing the distinct modes of action of SMFs and PEMFs is critical for optimizing magnetic stimulation strategies in regenerative medicine and bone tissue engineering.

It is important to note that while multiple studies employ magnetic stimulation to enhance osteogenic activity, there is no established consensus regarding the optimal frequency, intensity, or field gradient, creating a knowledge gap regarding the parameter space of magnetic stimulation. Various studies have utilized different magnetic field conditions, often selected empirically, and very few have systematically compared outcomes across variable field parameters. Furthermore, while magnetic stimulation is a promising strategy in bone tissue engineering, current research is predominantly conducted in in vitro settings, with a limited number of in vivo studies available. Therefore, magnetic field penetration depth, which is a critical factor for in vivo translation, has received limited attention. Low‐frequency fields are generally more capable of penetrating deeper tissues,^[^ ^90^ ^]^ but their biological impact remains underexplored. Future research should address these gaps by standardizing stimulation protocols and investigating how magnetic field characteristics affect tissue penetration and therapeutic potential in disease‐relevant in vivo models. Furthermore, with respect to cell and scaffold interactions via magnetic field applications, it was observed that when both cells and scaffolds are magnetized (MC+MS), cell‐scaffold affinity is improved due to magnetically mediated attraction, further promoting adhesion and integration.^[^ ^91^ ^]^

### 4.5 Considerations of Toxicity and Cell Type Diversity

The long‐term cytotoxicity of MPs, particularly iron oxide nanoparticles, remains a concern. Key factors influencing toxicity include: iron oxidation state, which can facilitate reactive oxygen species (ROS) production,^[^ ^49^ ^]^ particle aggregation, and dosage levels.^[^ ^32^ ^]^

Most studies to date have focused on osteoblasts or mesenchymal stem cells, with limited investigation into other cell types. Expanding research to include endothelial cells or immune cells would offer a more complete understanding of the multicellular interactions occurring in magnetically stimulated environments.

### 4.6 Perspective

MPs have also shown promise in scaffold deformation, cell patterning, and the creation of dynamic, responsive constructs. For example, magnetic actuation has been used to stimulate mechanical deformation in scaffolds,^[^ ^80^ ^]^ and inhomogeneous magnetic gradients enable precise cell positioning within complex architectures.^[^ ^85^ ^]^ However, the full potential of these techniques, especially in replicating native bone microarchitectures, remains underexplored. Future research should aim to: broaden the range of magnetic field parameters applied during cell culture, determine if MP‐induced osteogenesis is purely magnetic or also related to particle size and surface effects (e.g., comparing superparamagnetic vs ferromagnetic behavior), develop hybrid fabrication methods (e.g., combining extrusion‐based 3D printing with electrospinning) to balance structural precision and fibrous texture,^[^ ^75^, ^92^ ^]^ investigate multi‐tissue interface engineering, such as bone‐cartilage or bone‐vascular constructs, using regionally magnetized scaffolds, perform in vitro and in vivo studies to understand MP behavior in physiological environments and to assess scaffold integration and biodegradation over time.

In summary, Figures 4 and 5 illustrate the key signaling pathways involved in mechanotransduction, highlighting the primary proteins that are upregulated or downregulated in response. Additionally, additional research is needed to: *i*) explore the impact of field intensity, frequency, and duration, *ii*) distinguish between intrinsic MPs effects and externally applied field effects, and *iii*) investigate how magnetic gradients and magnetic field penetration depths generated near cells contribute to enhanced signaling.^[^ ^60^ ^]^

Magnetized systems, whether applied to scaffolds, cells, or both, provide a highly adaptable platform for enhancing bone regeneration through mechanical and biological cues. While considerable progress has been made, the field now requires standardization, mechanistic studies, and multicellular in vitro and in vivo models to transition from proof‐of‐concept to clinical application. The integration of biomaterials science, biomechanics, and tissue biology will be key to fully realizing the potential of magnetic particle‐based strategies in tissue engineering and regenerative medicine.

## 5 Conclusion

Recent advances in bone tissue engineering have increasingly highlighted the potential of MPs to enhance scaffold functionality and stimulate cellular responses. This review discusses current knowledge on the synthesis of MPs, the fabrication of MSs, and the MCs, along with the combined MC + MS approach. The biological effects of MPs were also discussed in the context of externally applied magnetic fields, which further amplify the inherent stimulatory effects of magnetized systems.

The choice of biomaterials and scaffold fabrication techniques plays a critical role in determining the mechanical and biological performance of MSs. Across studies, the incorporation of MPs has been shown to improve scaffold mechanical strength (e.g., compressive modulus, toughness), increase surface roughness, enhance cell adhesion, and support osteogenic differentiation in various osteoprogenitors and stem cells. When exposed to static or alternating magnetic fields, these effects are further enhanced through mechanisms involving magnetic field gradients, mechanotransduction, and activation of intracellular signaling pathways such as MAPK and BMP/Smads. These findings collectively support the creation of a favorable biophysical microenvironment that promotes bone regeneration through non‐contact magnetic stimulation. Importantly, the combination of MCs and MSs allows for synergistic interactions, wherein magnetized cells exhibit enhanced adhesion to magnetized scaffolds, improving integration and tissue organization.

Considering the above‐mentioned points, this strategy (i.e., using MPs and external magnetic fields) represents a promising, adaptable, and non‐invasive platform for stimulating bone repair and regeneration. Continued optimization of MP formulations, scaffold fabrication methods, and magnetic field parameters will be essential for advancing these systems toward preclinical and clinical applications in tissue engineering and regenerative medicine.

## Conflict of Interest

The authors declare no conflict of interest.

## References

[1] N. Xue , X. Ding , R. Huang , R. Jiang , H. Huang , X. Pan , W. Min , J. Chen , J.‐A. Duan , Liu , Y. Wang , Pharmaceuticals (Basel) 2022, 15, 879. 35890177 10.3390/ph15070879 PMC9324138

[2] A. Nauth , E. Schemitsch , B. Norris , Z. Nollin , J. T. Watson , J. Ortho. Trauma 2018, 32, S7. 10.1097/BOT.0000000000001115 29461395

[3] G. Paltanea , V. Manescu Paltanea , I. Antoniac , A. Antoniac , I. V. Nemoianu , A. Robu , H. Dura , Int. J. Mol. Sci. 2023, 24, 4312. 36901743 10.3390/ijms24054312 PMC10001544

[4] A. Dasari , J. Xue , S. Deb , Nanomaterials 2022, 12, 757. 35269245 10.3390/nano12050757 PMC8911835

[5] T. Zhu , H. Zhou , X. Chen , Y. Zhu , Front. Bioeng. Biotechnol. 2023, 11, 2023. 10.3389/fbioe.2023.1296881 PMC10691504 38047283

[6] T. P. Ribeiro , M. Flores , S. Madureira , F. Zanotto , F. J. Monteiro , M. S. Laranjeira , Pharmaceutics 2023, 15, 1045. 37111531 10.3390/pharmaceutics15041045 PMC10143200

[7] Z. Li , L. Xue , Wang , X. Ren , Y. Zhang , C. Wang , J. Sun , Materials (Basel, Switzerland) 2023, 16, 1429. 36837058 10.3390/ma16041429 PMC9961196

[8] N. Bock , A. Riminucci , C. Dionigi , A. Russo , A. Tampieri , E. Landi , V. A. Goranov , M. Marcacci , V. Dediu , Acta Biomater. 2010, 6, 786. 19788946 10.1016/j.actbio.2009.09.017

[9] A. Gloria , T. Russo , U. D'Amora , S. Zeppetelli , T. D'Alessandro , M. Sandri , M. Bañobre‐López , Y. Piñeiro‐Redondo , M. Uhlarz , A. Tampieri , J. Rivas , T. Herrmannsdörfer , V. A. Dediu , L. Ambrosio , R. De Santis , J. R. Soc. Interface 2013, 10, 20120833. 23303218 10.1098/rsif.2012.0833 PMC3565733

[10] M. J. Page , J. E. McKenzie , M. Bossuyt , I. Boutron , T. C. Hoffmann , C. D. Mulrow , L. Shamseer , J. M. Tetzlaff , E. A. Akl , S. E. Brennan , R. Chou , J. Glanville , J. M. Grimshaw , A. Hróbjartsson , M. M. Lalu , T. Li , E. W. Loder , E. Mayo‐Wilson , S. McDonald , L. A. McGuinness , L. A. Stewart , J. Thomas , A. C. Tricco , V. A. Welch , Whiting , D. Moher , BMJ 2021, 372, n71. 33782057 10.1136/bmj.n71 PMC8005924

[11] D. Fayol , N. Luciani , L. Lartigue , F. Gazeau , C. Wilhelm , Adv. Healthcare Mater. 2013, 2, 313. 10.1002/adhm.201200294 23184893

[12] H. Sadeghzadeh , A. Mehdipour , H. Dianat‐Moghadam , R. Salehi , A. B. Khoshfetrat , A. Hassani , D. Mohammadnejad , Stem Cell Res. Ther. 2022, 13, 143. 35379318 10.1186/s13287-022-02816-0 PMC8981929

[13] J. R. Henstock , M. Rotherham , A. J. El Haj , J. Tissue Eng. 2018, 9, 2041731418808695. 30397432 10.1177/2041731418808695 PMC6207961

[14] T. S. S. Carvalho , M. C. Torres , J. H. Belo , J. Mano , S. M. Olhero , Adv. NanoBiomed Res. 2023, 3, 2023.

[15] J. Ge , R. Asmatulu , B. Zhu , Q. Zhang , S.‐Y. Yang , Bioengineering (Basel) 2022, 9, 278. 35877329 10.3390/bioengineering9070278 PMC9311534

[16] S. Sahmani , A. Khandan , S. Saber‐Samandari , M. M. Aghdam , Mater. Sci. Eng. C Mater. Biol. Appl. 2020, 111, 110835. 32279734 10.1016/j.msec.2020.110835

[17] G. Calabrese , S. Petralia , C. Fabbi , S. Forte , D. Franco , S. Guglielmino , E. Esposito , S. Cuzzocrea , F. Traina , S. Conoci , Regen. Biomater. 2020, 7, 461. 33149935 10.1093/rb/rbaa033 PMC7597806

[18] A. Ito , M. Kamihira , Prog. Mol. Biol. Transl. Sci. 2011, 104, 355. 22093224 10.1016/B978-0-12-416020-0.00009-7

[19] S. I. Mostafa , M. S. S. Ismail , H. A. A. Mohammed , M. F. F. Osman , N. A. A. Elwassefy , Emerg. Mater. 2023, 6, 1273.

[20] A. Pekarsky , O. Spadiut , Front. Bioeng. Biotechnol. 2020, 8, 573183. 33195134 10.3389/fbioe.2020.573183 PMC7604359

[21] L. B. Kong , L. Liu , Z. Yang , S. Li , T. Zhang , C. Wang , in Magnetic, Ferroelectric, and Multiferroic Metal Oxides (Ed: B. D. Stojanovic ), Elsevier, Amsterdam, Netherlands 2018.

[22] T. Kim , M. Shima , J. Appl. Phys. 2007, 101, 09M516.

[23] A. Hubert , R. Schäfer , Magnetic Domains: The Analysis of Magnetic Microstructures, Springer Science & Business Media, Berlin, Germany 1998.

[24] Q. Li , C. W. Kartikowati , S. Horie , T. Ogi , T. Iwaki , K. Okuyama , Sci. Rep. 2017, 7, 9894. 28855564 10.1038/s41598-017-09897-5 PMC5577113

[25] K. Ali , A. K. Sarfraz , I. M. Mirza , A. Bahadur , S. Iqbal , A. ul Haq , Curr. Appl. Phys. 2015, 15, 925.

[26] K. Wu , D. Su , J. Liu , R. Saha , J.‐P. Wang , Nanotechnology 2019, 30, 502003. 31491782 10.1088/1361-6528/ab4241

[27] K. Mylkie , Nowak , Rybczyński , M. Ziegler‐Borowska , Materials 2021, 14, 248. 33419055 10.3390/ma14020248 PMC7825442

[28] N. Ajinkya , X. Yu , Kaithal , H. Luo , Somani , S. Ramakrishna , Materials 2020, 13, 4644. 33080937 10.3390/ma13204644 PMC7603130

[29] H.‐M. Yun , S.‐J. Ahn , K.‐R. Park , M.‐J. Kim , J.‐J. Kim , G.‐Z. Jin , H.‐W. Kim , E.‐C. Kim , Biomaterials 2016, 85, 88. 26854394 10.1016/j.biomaterials.2016.01.035

[30] X. Sun , Y. Gao , Z. Li , J. He , Y. Wu , Biomaterials 2023, 295, 122051. 36812842 10.1016/j.biomaterials.2023.122051

[31] D. Bokov , A. Turki Jalil , S. Chupradit , W. Suksatan , M. Javed Ansari , I. H. Shewael , G. H. Valiev , E. Kianfar , Adv. Mater. Sci. Eng. 2021, 2021, 5102014.

[32] A. P. Sangnier , A. B. Van de Walle , A. Curcio , R. Le Borgne , L. Motte , Y. Lalatonne , C. Wilhelm , Nanoscale 2019, 11, 16488. 31453605 10.1039/c9nr05624f

[33] M. J. Eskandari , I. Hasanzadeh , Mater. Sci. Eng., B 2021, 266, 115050.

[34] J. R. Perez , D. Kouroupis , D. J. Li , T. M. Best , L. Kaplan , D. Correa , Front. Bioeng. Biotechnol. 2018, 6, 2018. 10.3389/fbioe.2018.00105 PMC6079270 30109228

[35] J. Huang , D. Wang , J. Chen , W. Liu , L. Duan , W. You , W. Zhu , J. Xiong , D. Wang , Saudi Pharm J 2017, 25, 575. 28579894 10.1016/j.jsps.2017.04.026 PMC5447436

[36] F. Sadeghian‐Nodoushan , H. Nikukar , M. Soleimani , A. Jalali‐Jahromi , S. Hosseinzadeh , A. Khojasteh , Iran J. Basic Med. Sci. 2022, 25, 1123. 36246059 10.22038/IJBMS.2022.64682.14237 PMC9526893

[37] M. Shokouhimehr , A. S. Theus , A. Kamalakar , L. Ning , C. Cao , M. L. Tomov , J. M. Kaiser , S. Goudy , N. J. Willett , H. W. Jang , C. N. LaRock , Hanna , A. Lechtig , M. Yousef , J. D. S. Martins , A. Nazarian , M. B. Harris , M. Mahmoudi , V. Serpooshan , Polymers 1099, 13, 1099. 10.3390/polym13071099 PMC8036866 33808295

[38] H.‐F. Liang , Y.‐P. Zou , A.‐N. Hu , B. Wang , J. Li , L. Huang , W.‐S. Chen , D.‐H. Su , L. Xiao , Y. Xiao , Y.‐Q. Ma , X.‐L. Li , L.‐B. Jiang , J. Dong , Adv. Healthcare Mater. 2023, 12, 2301724. 10.1002/adhm.202301724 37767893

[39] A. Yadav , M. Bagade , S. Ghumnani , S. Raman , B. Saha , K. Kubatzky , R. Ashma , Biol. Chem. 2021, 403, 1. 34882360 10.1515/hsz-2021-0290

[40] S. Behzadi , V. Serpooshan , W. Tao , M. A. Hamaly , M. Y. Alkawareek , E. C. Dreaden , D. Brown , A. M. Alkilany , O. C. Farokhzad , M. Mahmoudi , Chem. Soc. Rev. 2017, 46, 4218. 28585944 10.1039/c6cs00636a PMC5593313

[41] X. Zhou , Y. Shi , L. Ren , S. Bao , Y. Han , S. Wu , H. Zhang , L. Zhong , Q. Zhang , J. Solid State Chem. 2012, 196, 138.

[42] M. Khalili , H. Keshvari , R. Imani , A. N. Sohi , E. Esmaeili , M. Tajabadi , Polym. Adv. Technol. 2022, 33, 782.

[43] Z. Jiao , Z. Deng , Y. Zhang , J. Wang , Polym. Bull. 2023, 81, 5627.

[44] F. D. Cojocaru , V. Balan , M. I. Popa , A. Lobiuc , A. Antoniac , I. V. Antoniac , L. Verestiuc , Int. J. Biol. Macromol. 2019, 125, 612. 30537500 10.1016/j.ijbiomac.2018.12.083

[45] I. Unalan , I. Occhipinti , M. Miola , E. Vernè , A. R. Boccaccini , Macromol. Biosci. 2023, 24, 2300397. 10.1002/mabi.202300397 PMC13420971 37902248

[46] M. Filippi , B. Dasen , J. Guerrero , F. Garello , G. Isu , G. Born , M. Ehrbar , I. Martin , A. Scherberich , Biomaterials 2019, 223, 119468. 31505394 10.1016/j.biomaterials.2019.119468

[47] S. K. Samal , V. Goranov , M. Dash , A. Russo , T. Shelyakova , Graziosi , L. Lungaro , A. Riminucci , M. Uhlarz , M. Bañobre‐López , J. Rivas , T. Herrmannsdörfer , J. Rajadas , S. De Smedt , K. Braeckmans , D. L. Kaplan , V. A. Dediu , ACS Appl. Mater. Interfaces 2015, 7, 23098. 26451743 10.1021/acsami.5b06813 PMC4867029

[48] H. Chen , J. Sun , Z. Wang , Y. Zhou , Z. Lou , B. Chen , Wang , Z. Guo , H. Tang , J. Ma , Y. Xia , N. Gu , F. Zhang , ACS Appl. Mater. Interfaces 2018, 10, 44279. 30499649 10.1021/acsami.8b17427

[49] P. Kush , Kumar , R. Singh , A. Kaushik , Asian J Pharm. Sci. 2021, 16, 704. 35027950 10.1016/j.ajps.2021.05.005 PMC8737424

[50] D. Lu , X. Wu , W. Wang , C. Ma , B. Pei , S. Wu , J. Nanomater. 2021, 3762490.

[51] J. Peuzin , Encyclopedia of Materials: Science and Technology, Elsevier, Amsterdam, Netherlands 2001.

[52] Y. Xia , Y. Guo , Z. Yang , H. Chen , K. Ren , M. D. Weir , L. C. Chow , M. A. Reynolds , F. Zhang , N. Gu , H. H. K. Xu , Mater. Sci. Eng. C Mater. Biol. Appl. 2019, 104, 109955. 31500064 10.1016/j.msec.2019.109955

[53] Q. Wang , B. Chen , F. Ma , S. Lin , M. Cao , Y. Li , N. Gu , Nano Res. 2017, 10, 626.

[54] X. Zhang , K. Yarema , A. Xu , Biological Effects of Static Magnetic Fields, Springer, Berlin, Germany 2017.

[55] Y. Xia , J. Sun , L. Zhao , F. Zhang , X.‐J. Liang , Y. Guo , M. D. Weir , M. A. Reynolds , N. Gu , H. H. K. Xu , Biomaterials 2018, 183, 151. 30170257 10.1016/j.biomaterials.2018.08.040

[56] H.‐M. Huang , S.‐Y. Lee , W.‐C. Yao , C.‐T. Lin , C.‐Y. Yeh , Clin. Orthop. Relat. Res. 2006, 447, 201. 16456312 10.1097/01.blo.0000203464.35561.be

[57] H. Kotani , H. Kawaguchi , T. Shimoaka , M. Iwasaka , S. Ueno , H. Ozawa , K. Nakamura , K. Hoshi , J. Bone Miner. Res. 2002, 17, 1814. 12369785 10.1359/jbmr.2002.17.10.1814

[58] J. Zhang , C. Ding , L. Ren , Y. Zhou , Shang , Prog. Biophys. Molecul. Biol. 2014, 114, 146. 10.1016/j.pbiomolbio.2014.02.001 24556024

[59] Y. Xia , H. Chen , Y. Zhao , F. Zhang , X. Li , L. Wang , M. D. Weir , J. Ma , M. A. Reynolds , N. Gu , H. H. K. Xu , Mater. Sci. Eng. C Mater. Biol. Appl. 2019, 98, 30. 30813031 10.1016/j.msec.2018.12.120

[60] V. Zablotskii , T. Polyakova , O. Lunov , A. Dejneka , Sci. Rep. 2016, 6, 37407. 27857227 10.1038/srep37407 PMC5114642

[61] M. M. Salmani , M. Hashemian , A. Khandan , Ceram. Int. 2020, 46, 27299.

[62] N. H. A. Ngadiman , Z. Zulkifli , N. M. Yusof , A. Idris , A. Z. A. Kadir , J. Pusppanathan , AIP Conf. Proc. 2019, 2129, 020035.

[63] E. Daskalakis , B. Huang , C. Vyas , A. A. Acar , A. Fallah , G. Cooper , A. Weightman , B. Koc , G. Blunn , Bartolo , Polymers 2022, 14, 445. 35160435 10.3390/polym14030445 PMC8839207

[64] E. Diaz , M. B. Valle , S. Ribeiro , S. Lanceros‐Mendez , J. M. Barandiaran , Materials 2019, 12, 3843. 31766520 10.3390/ma12233843 PMC6926959

[65] S. Scialla , A. Barca , B. Palazzo , U. D'Amora , T. Russo , A. Gloria , R. De Santis , T. Verri , A. Sannino , L. Ambrosio , F. Gervaso , J. Biomed. Mater. Res. A 2019, 107, 1244. 30701656 10.1002/jbm.a.36633

[66] E. Tanasa , C. Zaharia , A. Hudita , I.‐C. Radu , M. Costache , B. Galateanu , Mater. Sci. Eng. C Mater. Biol. Appl. 2020, 110, 110714. 32204026 10.1016/j.msec.2020.110714

[67] P. Niemeyer , U. Krause , J. Fellenberg , Kasten , A. Seckinger , A. D. Ho , H.‐G. Simank , Cells Tissues Organs 2004, 177, 68. 15297781 10.1159/000079182

[68] Y. Zhu , Q. Yang , M. Yang , X. Zhan , F. Lan , J. He , Z. Gu , Y. Wu , ACS Nano 2017, 11, 3690. 28314099 10.1021/acsnano.6b08193

[69] J.‐J. Kim , R. K. Singh , S.‐J. Seo , T.‐H. Kim , J.‐H. Kim , E.‐J. Lee , H.‐W. Kim , RSC Adv. 2014, 4, 17325.

[70] Y. Zhu , Z. Li , Y. Zhang , F. Lan , J. He , Y. Wu , Nanoscale 2020, 12, 8720. 32285072 10.1039/d0nr00867b

[71] M. B. Frank , S. E. Naleway , T. Haroush , C.‐H. Liu , S. H. Siu , J. Ng , I. Torres , A. Ismail , K. Karandikar , M. M. Porter , O. A. Graeve , J. McKittrick , Mater. Sci. Eng. C Mater. Biol. Appl. 2017, 77, 484. 28532056 10.1016/j.msec.2017.03.246

[72] R. Govindan , S. Karthi , G. S. Kumar , E. K. Girija , Mater. Adv. 2020, 1, 3466.

[73] C. J. Mortimer , C. J. Wright , Biotechnol. J. 2017, 12, 1600693. 10.1002/biot.201600693 28635132

[74] Y. Yu , S. Ren , Y. Yao , H. Zhang , C. Liu , J. Yang , W. Yang , L. Miao , J. Biomed. Nanotechnol. 2017, 13, 835. 10.1166/jbn.2017.2451 29490760

[75] Y. yu , S. Hua , M. Yang , Z. Fu , S. Teng , K. Niu , Q. Zhao , C. Yi , RSC Adv. 2016, 6, 110557.

[76] S. H. Huang , Liu , A. Mokasdar , L. Hou , Int. J. Adv. Manuf. Technol. 2013, 67, 1191.

[77] F. J. T. M. Tavares , I. P. Soares , J. C. Silva , J. P. Borges , Int. J. Mol. Sci. 2023, 24, 1128. 36674644 10.3390/ijms24021128 PMC9863008

[78] C.‐Y. Kao , T.‐L. Lin , Y.‐H. Lin , A. K.‐X. Lee , S. Y. Ng , T.‐H. Huang , T.‐T. Hsu , Cells 2022, 11, 3967.36552731

[79] A. Yang , Y. Wang , Q. Feng , K. Fatima , Q. Zhang , X. Zhou , C. He , Adv. Healthcare Mater. 2024, 13, 2302687. 10.1002/adhm.202302687 37940192

[80] J. Spangenberg , D. Kilian , C. Czichy , T. Ahlfeld , A. Lode , S. Günther , S. Odenbach , M. Gelinsky , ACS Biomater. Sci. Eng. 2021, 7, 648. 33507748 10.1021/acsbiomaterials.0c01371

[81] C. Czichy , D. Kilian , T.‐C. Wang , S. Günther , A. Lode , M. Gelinsky , S. Odenbach , J. Mech. Behav. Biomed. Mater. 2022, 131, 105253. 35490511 10.1016/j.jmbbm.2022.105253

[82] H.‐Y. Xu , N. Gu , Front. Mater. Sci. 2014, 8, 20.

[83] Basic and Applied Bone Biology (Eds: D. Burr , M. Allen ), Academic Press, Cambridge, Massachusetts 2013.

[84] C. Czichy , S. Odenbach , Smart Mater. Struct. 2023, 32, 115008.

[85] V. Goranov , T. Shelyakova , R. De Santis , Y. Haranava , A. Makhaniok , A. Gloria , A. Tampieri , A. Russo , E. Kon , M. Marcacci , L. Ambrosio , V. A. Dediu , Sci. Rep. 2020, 10, 2289. 32041994 10.1038/s41598-020-58738-5 PMC7010825

[86] J. Simińska‐Stanny , M. Nizioł , Szymczyk‐Ziółkowska , M. Brożyna , A. Junka , A. Shavandi , D. Podstawczyk , Addit. Manuf. 2022, 49, 102506.

[87] R. Xie , Y. Cao , R. Sun , R. Wang , A. Morgan , J. Kim , S. J. P. Callens , K. Xie , J. Zou , J. Lin , K. Zhou , X. Lu , M. M. Stevens , Sci. Adv. 2024, 10, adl1549. 10.1126/sciadv.adl1549 PMC10836728 38306430

[88] C. L. Ross , Y. Zhou , C. E. McCall , S. Soker , T. L. Criswell , Bioelectricity 2019, 1, 247. 34471827 10.1089/bioe.2019.0026 PMC8370292

[89] A. Pilla , R. Fitzsimmons , D. Muehsam , J. Wu , C. Rohde , D. Casper , Biochim. Biophys. Acta 2011, 1810, 1236. 22005645 10.1016/j.bbagen.2011.10.001

[90] P. P. Ascona García , G. E. Ordoñez Carpio , W. M. Zelada Zamora , E. Villanueva Pedraza , R. A. Fernandez Villarroel , Appl. Sci. 2025, 15, 2225.

[91] M. Petretta , A. Gambardella , G. Desando , C. Cavallo , I. Bartolotti , T. Shelyakova , V. Goranov , M. Brucale , V. A. Dediu , M. Fini , B. Grigolo , Polymers 2021, 13, 3825. 34771382 10.3390/polym13213825 PMC8588077

[92] Y. Danlei , F. Fazal , J.‐X. Wang , N. Radacsi , Adv. Mater. Technol. 2022, 7, 2101309.

[93] B. Thavornyutikarn , N. Chantarapanich , K. Sitthiseripratip , G. Thouas , Q. Chen , Prog. Biomater. 2014, 3, 61. 26798575 10.1007/s40204-014-0026-7 PMC4709372

[94] Y.‐W. Ge , M. Chu , Z.‐Y. Zhu , Q.‐F. Ke , Y.‐P. Guo , C.‐Q. Zhang , W.‐T. Jia , Mater. Today Bio. 2022, 16, 100439. 10.1016/j.mtbio.2022.100439 PMC9557728 36245833

[95] A. Tampieri , T. D'Alessandro , M. Sandri , S. Sprio , E. Landi , L. Bertinetti , S. Panseri , G. Pepponi , J. Goettlicher , M. Bañobre‐López , J. Rivas , Acta Biomater. 2012, 8, 843. 22005331 10.1016/j.actbio.2011.09.032

[96] H.‐Y. Lin , H.‐Y. Huang , S.‐J. Shiue , J.‐K. Cheng , J. Magn. Magn. Mater. 2020, 504, 166680.

[97] A. Tampieri , M. Iafisco , M. Sandri , S. Panseri , C. Cunha , S. Sprio , E. Savini , M. Uhlarz , T. Herrmannsdörfer , ACS Appl. Mater. Interfaces 2014, 6, 15697. 25188781 10.1021/am5050967

